# Mixed Methods Research on Family Caregiving for Stroke Survivors: A Methodological Systematic Review

**DOI:** 10.1111/jan.70534

**Published:** 2026-03-04

**Authors:** Tharaa Ananzeh, Reem Telfah, Tamilyn Bakas, Raghad Tawalbeh, Vicki L. Plano Clark

**Affiliations:** ^1^ College of Nursing University of Cincinnati Cincinnati Ohio USA; ^2^ College of Nursing Saginaw Valley State University Saginaw Michigan USA; ^3^ College of Nursing University of Wisconsin–Milwaukee Milwaukee Wisconsin USA; ^4^ School of Education University of Cincinnati Cincinnati Ohio USA

**Keywords:** caregivers, family caregiving, methodological review, mixed methods, stroke survivors

## Abstract

**Aim:**

To examine how mixed methods research has been applied in studies of family caregiving for stroke survivors, focusing on key methodological components (rationale, design types, integration strategies, and use of joint displays).

**Design:**

Methodological systematic review.

**Methods:**

A systematic search of five databases yielded 17 studies. The extraction focused on mixed methods features (rationale, design, integration, joint displays), and quality was appraised using the Mixed Methods Appraisal Tool.

**Data Sources:**

PubMed, CINAHL, Scopus, Web of Science, and PsycINFO were searched for relevant studies published from 2010 to 2025.

**Results:**

The included studies addressed topics such as caregiver burden, coping, resilience, and intervention outcomes. Convergent and explanatory sequential designs predominated. Complementarity was the most frequent rationale for mixing methods. Integration occurred mainly through merging, with fewer instances of connecting or building. Three studies included joint displays to integrate the results.

**Conclusion:**

Mixed methods research is increasingly applied in family caregiving. To advance the field, researchers should strengthen integration during analysis and results and improve transparency in reporting key design features.

**Implications for the Profession and/or Patient Care:**

Strengthening methodological rigour in mixed methods studies on stroke caregiving will improve the evidence base for nursing practice. Intentional and meaningful integration of qualitative and quantitative evidence can better inform effective interventions and support programs, ultimately enhancing care for stroke survivors and their families.

**Impact:**

This review evaluates how mixed methods research is applied in family caregiving studies. It identifies significant methodological gaps, including unclear reporting of design and limited use of advanced integration techniques. The recommendations provide practical guidance for researchers to improve reporting and integration, yielding richer evidence to inform interventions and policies that support family caregivers.

**Reporting Method:**

The review followed the PRISMA 2021 guidelines for transparent reporting of systematic reviews.

**Patient or Public Contribution:**

No patient or public involvement.

## Summary

- What does this paper contribute to the wider global clinical community?
  ○ Provides the first methodological systematic review examining how mixed methods research is applied in studies of family caregiving for stroke survivors, identifying prevailing designs, rationales, and integration strategies.
  ○ Highlights substantial gaps in methodological reporting, including limited use of advanced integration techniques and insufficient clarity in describing selected designs.
  ○ Offers targeted, evidence‐based recommendations to improve methodological rigour, transparency, and integration in future mixed methods studies, strengthening the global evidence base for caregiver support interventions.

## 1 Introduction

Stroke is recognized as a primary cause of long term disability in the United States (Martin et al. 2024). More than half of stroke survivors experience impaired mobility (Tsao et al. 2023) and face difficulties with essential daily activities, including walking, bathing, and feeding (Kalavina et al. 2019). Stroke survivors mainly depend on others to help them do essential daily activities (Poomalai et al. 2023). Providing care for a stroke survivor is challenging, as caregivers often take on responsibilities for both personal care and emotional support (American Heart Association 2023). This caregiving role can directly affect the physical, emotional, and psychosocial well‐being of caregivers (Choo et al. 2023; Kokorelias et al. 2020). Although they face these significant challenges and demands, they strive to provide compassionate care through resilience and effective coping strategies (Wang et al. 2023).

Given the multifaceted nature of stroke caregiving, a comprehensive understanding of caregivers' experiences requires innovative research methodologies. However, much of what is currently understood about family caregiving for stroke survivors has been derived primarily from either quantitative or qualitative studies, often in isolation. For instance, Kokorelias et al. (2020) conducted a qualitative meta‐synthesis review to explore caregivers' experiences. On the other hand, Jammal et al. (2024) performed a quantitative systematic review to assess interventions that aimed to alleviate and reduce family caregivers' burden. This fragmented and separate approach limits the depth of the findings because quantitative data fail to capture personal lived experiences, while qualitative data offer an in‐depth perspective but limit the generalizability of results. To fully capture the complex and demanding nature of family caregiving, a more integrative research approach is needed. Mixed methods research offers a unique opportunity to integrate qualitative and quantitative methods within a single study to achieve a more comprehensive understanding of a research problem (Johnson et al. 2007). It involves the collection and analysis of both qualitative and quantitative data as well as the integration of their results to address research questions effectively (Creswell and Plano Clark 2018). However, the potential of mixed methods research to advance understanding of stroke caregiving will only be realised if researchers employ high‐quality strategies that achieve meaningful integration of qualitative and quantitative strands.

To ensure high‐quality mixed methods research, researchers must give careful attention to essential components such as the rationale, the selected design, integration procedures, and the use of joint displays. Each of these elements has been elaborated in established methodological frameworks (Creswell and Plano Clark 2018; Fetters et al. 2013; Greene et al. 1989). The rationale is the intent and justification for mixing methods in a study; in other words, the reasons and purposes for using mixed methods research. Authors convey their rationale for adopting mixed methods research in the introduction, methods, or discussion (Greene et al. 1989). Mixed methods designs serve as prototypes or models that illustrate the different logics available for combining qualitative and quantitative approaches. Each design type has its own specific procedures for conducting and integrating methods to address a particular research purpose (Creswell and Plano Clark 2018). A critical aspect of mixed methods research is data integration, which is the explicit interconnection of quantitative and qualitative components within a mixed methods study to generate more comprehensive and meaningful insights (Fetters et al. 2013). One strategy to enhance integration is the use of joint displays. Joint displays are visual tools, such as tables and figures, that researchers develop to jointly analyse quantitative and qualitative data side by side, facilitate comparison, deepen interpretation, and illustrate how findings are connected (Guetterman et al. 2015). The quality of integration and whether it offers new insights remains one of the biggest challenges in mixed methods research (Fetters and Freshwater 2015).

## 2 The Review

Despite the growing use of mixed methods research in healthcare, no systematic review has synthesised how mixed methods research has been explicitly applied to family caregiving for stroke survivors. Previous mixed methods reviews have examined family caregiving research in other populations, such as family caregivers of patients undergoing dialysis (Hoang et al. 2018), caregivers of individuals with Alzheimer's disease (Gonella et al. 2022), and end‐of‐life (Morgan et al. 2020). However, these reviews mainly focused on caregiving outcomes rather than on the methodological aspects of mixed methods research. At the same time, stroke caregiving presents uniquely complex challenges that warrant focused methodological attention. Unlike other chronic conditions, stroke occurs suddenly and often leads to long‐term disability, cognitive changes, and unpredictable recovery, placing sustained demands on family caregivers (Tsao et al. 2023; Kokorelias et al. 2020). These challenges make stroke caregiving a uniquely important context for evaluating how mixed methods are applied. As a result, little is known about how mixed methods research is being used to study stroke caregiving, what specific mixed methods research designs are most common, how data integration is handled, and what rationales researchers provide for using mixed methods research. Understanding how mixed methods research has been applied in stroke caregiving enables researchers to learn from existing strengths and limitations, identify methodological exemplars, and inform the design of future studies that achieve more rigorous and meaningful integration.

## 3 Aim(s)

The purpose of this review is to systematically examine the methodological application of mixed method research in studies on family caregiving for stroke survivors. Thus, this review aims to address the following research questions:

1. What topics and purposes have been examined in studies of family caregiving for stroke survivors using mixed methods research?
2. What types of qualitative and quantitative data have been used to address these topics and purposes?
3. How do researchers approach mixed method research components, such as rationale, design, integration, and joint display use, in studies on family caregiving for stroke survivors?

## 4 Methods

### 4.1 Design

We conducted a systematic methodological review, which is defined as a type of review that formally examines existing literature with a focus on methodological issues. It synthesises and critically appraises practices related to research methods, summarises current approaches, and provides evidence‐based recommendations to improve methodological practice (Aguinis et al. 2020). Mixed methods systematic methodological reviews can contribute to advancing methodological practice in several ways (Howell Smith and Shanahan Bazis 2021). They describe practices and trends, highlight the prevalence of mixed methods in specific disciplines, and identify problematic practices such as a lack of explicit rationale or inadequate integration of strands. At the same time, they showcase methodological exemplars and encourage innovative approaches, including hybrid forms of data collection and novel strategies for integrating qualitative and quantitative findings. Together, these contributions strengthen the quality and impact of future mixed method research (Fàbregues and Guetterman 2025). This review aimed to synthesise how mixed method research has been applied in the context of family caregiving for stroke survivors. Rather than evaluating caregiving outcomes, it focused on analysing how researchers implemented and reported key mixed method research components, including the rationale for using mixed method research, the design, integration strategies, and the use of joint displays. This review was guided by established methodological frameworks. For rationale classification, Greene et al. (1989); for design typologies, Creswell and Plano Clark (2018); and for integration approaches, Fetters et al. (2013). These frameworks guided both data extraction and synthesis in this review.

### 4.2 Search Methods

A literature search was conducted across five databases: PubMed, CINAHL, Scopus, Web of Science, and PsycINFO. These databases were chosen because they collectively provide comprehensive coverage of health, nursing, behavioural science, and multidisciplinary research relevant to mixed methods studies on family caregiving. The search strategy incorporated both Medical Subject Headings (MeSH) and keywords to identify relevant studies on family caregiving for stroke survivors. Keywords included family caregiver, stroke caregiver, informal caregiver, stroke survivor, mixed methods, multi method, mixed research, mixed model research, and integrated research. Boolean operators (AND, OR) were applied to refine and enhance the search results. The complete search strings for each database are presented in Table 1.

**TABLE 1:** Search strategy.

Database	Search string
PubMed	(“Stroke”[Mesh] OR stroke[tiab] OR “stroke survivors”[tiab] OR “cerebrovascular accident”[tiab] OR “brain attack”[tiab]) AND (“Caregivers”[Mesh] OR caregiver*[tiab] OR “family caregiving”[tiab] OR “informal caregiver”[tiab] OR “family carer”[tiab]) AND (“Mixed Methods”[tiab] OR “Mixed Method”[tiab] OR “mixed‐methods”[tiab] OR “multi‐method”[tiab] OR “multimethod”[tiab] OR “mixed research”[tiab] OR “integrated research”[tiab] OR “MMR”[tiab] OR “convergent design”[tiab] OR “exploratory sequential”[tiab] OR “explanatory sequential”[tiab])
CINAHL	(MH “Stroke+” OR “stroke” OR “stroke survivors” OR “cerebrovascular accident” OR “brain attack”) AND (MH “Caregivers+” OR “caregiver*” OR “family caregiving” OR “informal caregiver” OR “family carer”) AND (“mixed methods” OR “mixed method” OR “mixed‐methods” OR “multi‐method” OR “multimethod” OR “mixed research” OR “integrated research” OR “MMR” OR “convergent design” OR “exploratory sequential” OR “explanatory sequential”)
Scopus	(TITLE‐ABS‐KEY (“stroke” OR “stroke survivors” OR “cerebrovascular accident” OR “brain attack”)) AND (TITLE‐ABS‐KEY (“caregiver*” OR “family caregiver*” OR “family caregiving” OR “informal caregiver” OR “family carer”)) AND (TITLE‐ABS‐KEY (“mixed methods” OR “mixed method” OR “mixed‐methods” OR “multi‐method” OR “multimethod” OR “mixed research” OR “integrated research” OR “MMR” OR “convergent design” OR “exploratory sequential” OR “explanatory sequential”))
Web of Science	(“stroke” OR “stroke survivors” OR “cerebrovascular accident” OR “brain attack”) AND (“caregiver*” OR “family caregiving” OR “informal caregiver” OR “family carer”) AND (“mixed methods” OR “mixed method” OR “mixed‐methods” OR “multi‐method” OR “multimethod” OR “mixed research” OR “integrated research” OR “MMR” OR “convergent design” OR “exploratory sequential” OR “explanatory sequential”)
PsycINFO	(DE “Stroke” OR TI “stroke” OR AB “stroke” OR TI “stroke survivors” OR AB “stroke survivors” OR TI “cerebrovascular accident” OR AB “cerebrovascular accident” OR TI “brain attack” OR AB “brain attack”) AND (DE “Family Caregivers” OR DE “Caregivers” OR TI “caregiver*” OR AB “caregiver*” OR TI “family caregiving” OR AB “family caregiving” OR TI “informal caregiver” OR AB “informal caregiver” OR TI “family carer” OR AB “family carer”) AND (TI “mixed methods” OR AB “mixed methods” OR TI “mixed method” OR AB “mixed method” OR TI “mixed‐methods” OR AB “mixed‐methods” OR TI “multi‐method” OR AB “multi‐method” OR TI “multimethod” OR AB “multimethod” OR TI “mixed research” OR AB “mixed research” OR TI “integrated research” OR AB “integrated research” OR TI “MMR” OR AB “MMR” OR TI “convergent design” OR AB “convergent design” OR TI “exploratory sequential” OR AB “exploratory sequential” OR TI “explanatory sequential” OR AB “explanatory sequential”)

### 4.3 Inclusion and/or Exclusion Criteria

The search was limited to articles published between March 2010 and March 2025 to ensure a comprehensive review of studies that used mixed methods related to family caregiving for stroke survivors. The inclusion criteria for this review focused on studies that (1) examine family caregivers of stroke survivors, (2) utilised a mixed methods approach that was explicitly identified by the authors as mixed methods research, (3) are published in peer‐reviewed journals, and (4) are written in English. Articles that focus on paid caregivers (e.g., nurses, physicians), review papers, books, dissertations, discussion papers, or research protocols were excluded. Additionally, studies focusing on family caregivers of individuals with conditions other than stroke (e.g., dementia, cancer, or traumatic brain injury) were excluded.

### 4.4 Search Outcome

**FIGURE 1:**
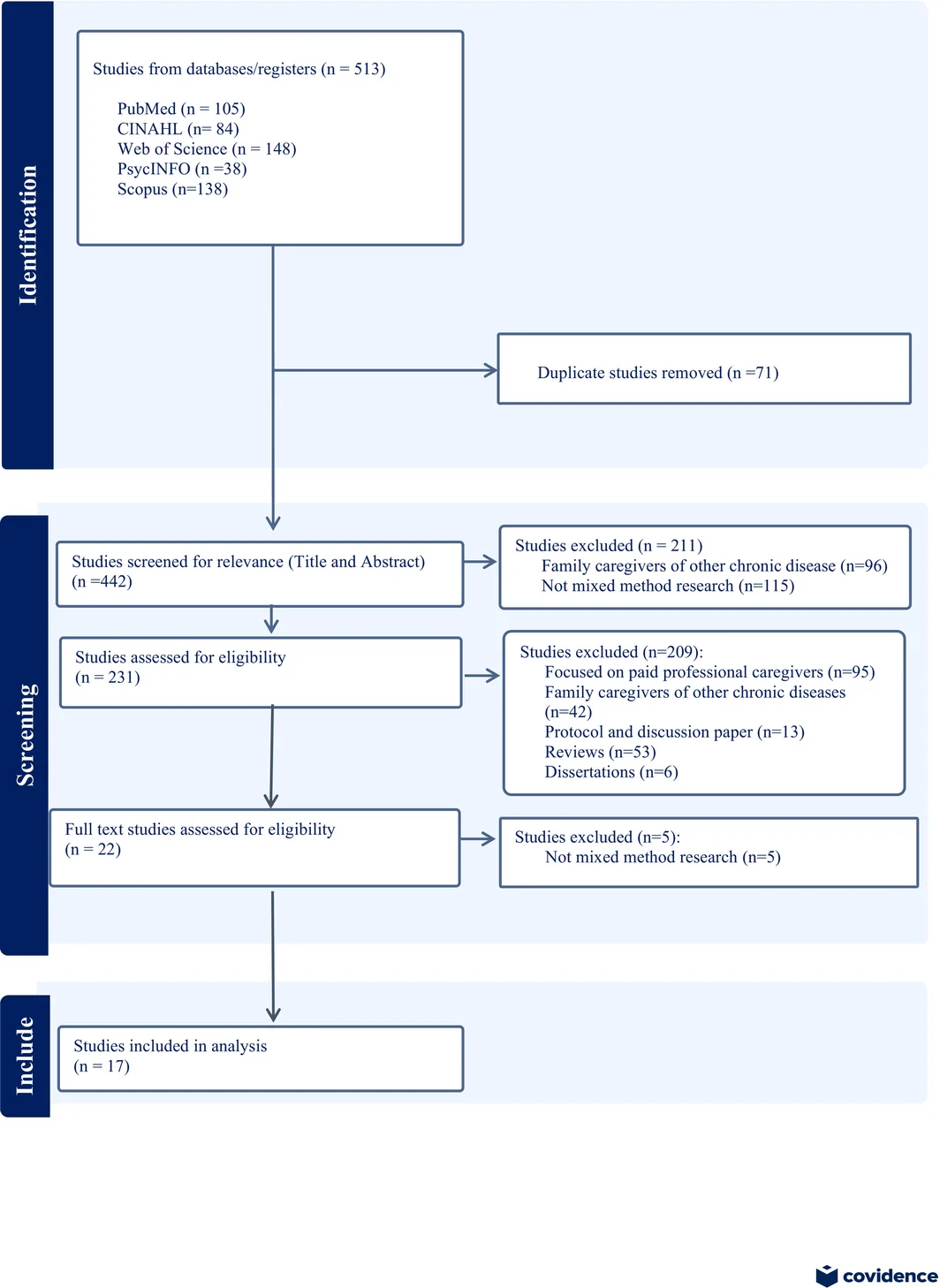
PRISMA flow chart.

Screening and study selection were managed using Covidence, a systematic review management software. A total of 513 records were retrieved from five databases: PubMed (*n* = 105), CINAHL (*n* = 84), Web of Science (*n* = 148), PsycINFO (*n* = 38), and Scopus (*n* = 138). Covidence automatically removed 71 duplicate records. The remaining 442 titles and abstracts were independently screened by two reviewers to assess eligibility, followed by full‐text review of 22 potentially relevant studies. A total of 120 articles were excluded as “not mixed methods.” Most of these were review papers (e.g., scoping, integrative, or systematic reviews) that mentioned mixed methods in their abstracts only to describe the designs of included studies, rather than applying a mixed methods approach themselves. In addition, because our search strategy intentionally incorporated terms such as *multi method* and *integrated research* to maximise sensitivity, we retrieved studies that used multi method to describe employing multiple qualitative methods or multiple quantitative measures, rather than integrating qualitative and quantitative strands. Finally, in line with our eligibility criteria, only empirical studies that explicitly utilised a mixed methods approach and were explicitly identified by the authors as mixed method research were retained. Any conflicts between the two reviewers were discussed and resolved by consensus. A total of 17 studies were included in this review. The screening process was illustrated using the Preferred Reporting Items for Systematic Reviews and Meta‐Analyses (PRISMA) framework (Page et al. 2021; See Figure 1). See Appendix S1 for the PRISMA checklist.

### 4.5 Quality Appraisal

The methodological quality of the included studies was evaluated using the Mixed Methods Appraisal Tool (MMAT; Hong et al. 2018). This validated tool is designed to assess studies across five methodological domains: qualitative (1.1–1.5), quantitative randomised controlled trials (2.1–2.5), quantitative non‐randomised studies (3.1–3.5), quantitative descriptive studies (4.1–4.5), and mixed methods research (5.1–5.5). Each appraisal begins with two general screening questions to determine whether the study is empirical and whether it contains sufficient methodological detail to proceed with the full appraisal. Studies that meet both screening criteria are then assessed using five quality criteria specific to their design type.

For mixed methods studies included in this review, we evaluated each component, qualitative, quantitative, and mixed methods, using the corresponding MMAT criteria, in accordance with the study's methodological structure. This approach ensured that the quality of all methodological strands was appraised systematically and independently. Two reviewers independently assessed each study. Any discrepancies in scoring were discussed to reach a consensus. Each criterion was rated as Y (Yes) if clearly met, N (No) if clearly not met, C (Cannot tell) if insufficient information was available, and (Not applicable) if the criterion did not apply to the study's design.

### 4.6 Data Abstraction

Data were extracted by two reviewers into two comprehensive tables that reflect both the caregiving focus and the methodological features of each study. The first table records the caregiving topics and study purposes, including: (1) the country in which the study was conducted, (2) the stated qualitative and quantitative purpose of the study, (3) the types of qualitative and quantitative data used, and (4) the key caregiving topics explored. These categories reflect Research Questions 1 and 2 by identifying the main topics and purposes in which mixed method research has been applied to family caregiving for stroke survivors and detailing the types of data collected across studies.

The second table captures the methodological application of mixed methods, including the mixed methods design (Creswell and Plano Clark 2018), the rationale for using mixed method research (Greene et al. 1989), the integration approach (Fetters et al. 2013), and the use of joint displays to enhance integration. According to Creswell and Plano Clark (2018), there are three core mixed method design types: convergent (concurrent) design, explanatory sequential design, and exploratory sequential design. In a convergent design, researchers collect quantitative and qualitative data separately but during the same phase of the study and then combine the results to compare or interpret them together. In an explanatory sequential design, researchers first collect and analyze quantitative data, and then collect qualitative data to help explain or expand on the quantitative findings. In an exploratory sequential design, researchers begin with qualitative data collection and analysis to explore a phenomenon, and then use the results to develop or inform a quantitative phase, such as a survey or intervention. In addition to these three core designs, Creswell and Plano Clark (2018) note that hybrid designs extend core models by combining them with other approaches, such as embedding a mixed methods component within a randomized controlled trial, case study, or program evaluation.

There are various rationales for using mixed method research, including triangulation for validity, complementarity for deeper interpretation, development to inform one method with another, initiation to uncover contradictions, and expansion to broaden the study's scope (Greene et al. 1989). The integration of the quantitative and qualitative aspects of a study provides the key advantage of mixed method research, but can be challenging to implement in practice (Fetters and Freshwater 2015). A useful framework for describing integration is that it can occur through connecting, building, and merging (Fetters et al. 2013). Connecting integration happens when the results of one strand are used to inform the sampling of the next strand. Building integration happens when the results of one strand are used to inform data collection methods in the next strand. Merging integration applies when the results of both strands are combined or co‐analysed together (Fetters et al. 2013). Joint displays are visual tools such as tables and figures used to jointly analyse quantitative and qualitative data side by side to enhance interpretations (Guetterman et al. 2015). The interpretation synthesises insights from both numerical data and participant narratives in the process of drawing inferences (Guetterman et al. 2015).

These methodological features directly reflect research question 3, which focuses on how researchers approach and implement key mixed method research components in studies of family caregiving. The chosen categories reflect the review's research questions and support the identification of patterns, gaps, and methodological strengths and limitations in the existing literature. To strengthen the quality of the methods, an expert reviewer in mixed methods research reviewed the procedures and verified the accuracy of the data extraction for nine (53%) of the included articles. This expert review provided an additional layer of quality assurance to strengthen the credibility of the review findings.

### 4.7 Synthesis

After the quality appraisal, evidence of the content and methods of studies was extracted and synthesised in three phases using qualitative content analysis (Schreier 2012). Phase one involved reviewing all included studies in full to establish familiarity with them. In phase two, we refined the data extraction process based on the initial thoughts that we developed from the familiarisation in phase one. Lastly, phase three entailed conducting data extraction for all included studies and comparing the methodological elements to identify patterns, similarities, and differences.

Descriptive and methodological synthesis strategies were employed. Key study characteristics, including country of origin and caregiving settings, were summarised through descriptive synthesis. This was followed by a methodological synthesis that examined how each study implemented key components of mixed methods research. Specifically, the synthesis focused on the stated rationale for using mixed methods, the design and methods employed, the data integration strategies, and whether joint displays were utilised to support integration.

## 5 Results

### 5.1 Characteristics of the Included Studies

Seventeen mixed method studies met the inclusion criteria, published between 2010 and 2025. These studies were conducted in diverse geographic contexts: seven in the United States (Caunca et al. 2020; Chen et al. 2016; Jordan et al. 2022; King et al. 2010; King et al. 2022; McCarthy and Lyons 2015; McCarthy et al. 2016) and five in Asia, specifically Indonesia (Na'imah et al. 2023), Malaysia (Rahman et al. 2024), Pakistan (Yousaf et al. 2019) and China (Han et al. 2024; Ye et al. 2024). Two studies were conducted in Canada (Cameron et al. 2015; Viscogliosi et al. 2019), one in the United Kingdom (Parkinson et al. 2022), one in Sweden (Ekstam et al. 2015), and one in Uganda (Eriksson et al. 2021). Most studies primarily focused on informal family caregivers of stroke survivors, although several also included stroke survivor–caregiver dyads as participants (Ekstam et al. 2015; McCarthy and Lyons 2015; McCarthy et al. 2016; Parkinson et al. 2022; Ye et al. 2024). The caregiving settings across the included studies varied widely, covering hospital wards, outpatient rehabilitation clinics, home environments, and community‐based programs. Some studies were conducted during the acute or rehabilitation phases within hospital settings, while others focused on caregivers providing long‐term support at home after discharge. Several studies explored caregiving in community‐based rehabilitation or support programs, and a few incorporated technology‐assisted interventions. See Table 3 for details.

### 5.2 Quality Appraisal Results

All 17 included studies met the initial screening criteria of the MMAT and were retained for full appraisal. Overall, most studies met the majority of relevant criteria for their respective designs. All the included studies fulfilled all five qualitative criteria (1.1–1.5). For the quantitative components, quality varied by study design. While most studies met the majority of quantitative criteria, several quantitative descriptive studies lacked sufficient reporting on sample representativeness and nonresponse bias. Additionally, some randomised and non‐randomised studies did not adequately describe methods used to control for confounding variables. The reporting quality of mixed methods components was generally adequate (5.1–5.5). Most studies provided a rationale for using a mixed methods design; however, one study did not mention any. Integration between qualitative and quantitative components was considered adequate, with some studies reporting it explicitly and others more implicitly. For addressing divergences between strands, most studies explained discrepancies when they occurred, although several were rated as “Cannot tell” due to insufficient reporting information. The weakest area was adherence to quality criteria for each methodological component (5.5), with some studies not fully meeting the standards for both the qualitative and quantitative strands. The detailed results of the quality appraisal are presented in Table 2.

**TABLE 2:** Quality appraisal of included studies (MMAT 2018 criteria).

Study (year)	Qualitative					Quantitative randomised control trials					Quantitative non‐randomised studies					Quantitative descriptive studies					Mixed methods				
	1.1	1.2	1.3	1.4	1.5	2.1	2.2	2.3	2.4	2.5	3.1	3.2	3.3	3.4	3.5	4.1	4.2	4.3	4.4	4.5	5.1	5.2	5.3	5.4	5.5
Rahman et al. (2024)	Y	Y	Y	Y	Y	—	—	—	—	—	—	—	—	—	—	Y	Y	Y	Y	Y	Y	Y	Y	Y	Y
Ye et al. (2024)	Y	Y	Y	Y	Y	—	—	—	—	—	—	—	—	—	—	Y	Y	Y	Y	Y	Y	Y	Y	Y	Y
Na'imah et al. (2023)	Y	Y	Y	Y	Y	—	—	—	—	—	—	—	—	—	—	Y	Y	Y	Y	Y	N	Y	Y	Y	Y
Jordan et al. (2022)	Y	Y	Y	Y	Y	—	—	—	—	—	C	Y	Y	N	Y	—	—	—	—	—	Y	Y	Y	Y	N
King et al. (2022)	Y	Y	Y	Y	Y	C	Y	Y	Y	Y	—	—	—	—	—	—	—	—	—	—	Y	Y	Y	Y	Y
Yousaf et al. (2019)	Y	Y	Y	Y	Y	—	—	—	—	—	—	—	—	—	—	Y	Y	Y	C	Y	Y	Y	Y	C	Y
King et al. (2010)	Y	Y	Y	Y	Y	—	—	—	—	—	—	—	—	—	—	Y	Y	Y	C	Y	Y	Y	Y	C	Y
Han et al. (2024)	Y	Y	Y	Y	Y	—	—	—	—	—	—	—	—	—	—	Y	Y	Y	Y	Y	Y	Y	Y	Y	Y
Viscogliosi et al. (2019)	Y	Y	Y	Y	Y	—	—	—	—	—	—	—	—	—	—	Y	C	Y	C	Y	Y	Y	Y	Y	Y
Ekstam et al. (2015)	Y	Y	Y	Y	Y	—	—	—	—	—	—	—	—	—	—	Y	C	Y	N	Y	Y	Y	Y	Y	N
Chen et al. (2016)	Y	Y	Y	Y	Y	—	—	—	—	—	—	—	—	—	—	Y	C	Y	C	Y	Y	Y	Y	Y	Y
Caunca et al. (2020)	Y	Y	Y	Y	Y	—	—	—	—	—	C	Y	Y	N	Y	—	—	—	—	—	Y	Y	Y	Y	N
McCarthy and Lyons (2015)	Y	Y	Y	Y	Y	—	—	—	—	—	—	—	—	—	—	Y	C	Y	C	Y	Y	Y	Y	Y	Y
Cameron et al. (2015)	Y	Y	Y	Y	Y	Y	Y	Y	Y	Y	—	—	—	—	—	—	—	—	—	—	Y	Y	Y	Y	Y
Eriksson et al. (2021)	Y	Y	Y	Y	Y	—	—	—	—	—	—	—	—	—	—	Y	C	Y	C	Y	Y	Y	Y	C	Y
McCarthy et al. (2016)	Y	Y	Y	Y	Y	—	—	—	—	—	—	—	—	—	—	Y	N	Y	C	Y	Y	Y	Y	C	N
Parkinson et al. (2022)	Y	Y	Y	Y	Y	—	—	—	—	—	C	Y	N	C	Y	—	—	—	—	—	Y	Y	Y	Y	N

*Note:* Criteria: 1.1–1.5 = qualitative; 2.1–2.5 = quantitative randomised; 3.1–3.5 = quantitative non‐randomised; 4.1–4.5 = quantitative descriptive; 5.1–5.5 = mixed methods. Y = Yes (criterion met), N = No (not met), C = Cannot tell, — = Not applicable (criterion does not apply to this study design).

### 5.3 Caregiving Topics, Purposes, and Data Types

This section presents the findings related to the caregiving topics studied in these mixed method studies, the purposes of the included studies, and the types of qualitative and quantitative data used (see Table 3).

**TABLE 3:** Caregiving topics and study purposes.

Author/year	Country	Caregiving Topics	Qualitative strand		Quantitative strand		
			Qualitative aim/question	Qualitative method used	Quantitative aim/question	Quantitative method used	Quantitative scales used
Cameron et al. (2015)	Canada	Family/friend caregivers of first‐time stroke survivors across acute and community care, participating in a phased support intervention program	To gather family caregivers' experiences and satisfaction with a new stroke family support program (Timing It Right)	In‐depth qualitative interviews and qualitative data from the recruitment and intervention delivery records	To pilot‐test the feasibility and preliminary effects of the Timing It Right Stroke Family Support Program on caregiver outcomes	Multi‐site randomised controlled trial (pilot)	20‐item Medical Outcomes Study Social Support Scale (Sherbourne and Stewart 1991): Positive Affect Scale (Watson et al. 1988); 20‐item Centre for Epidemiological Studies Depression Scale (CESD) (Radloff 1977); Pearlin's 7‐item Mastery Scale (Cameron et al. 2015); Stroke Knowledge Test (Sullivan and Dunton 2004); Caregiving Impact scale and Caregiving Assistance Scale (Cameron et al. 2002; Sullivan and Dunton 2004)
Caunca et al. (2020)	USA	Development of a web‐based support system for family caregivers aimed at reducing caregiver burden	To identify the needs, concerns, and feedback of stroke caregivers to guide the development of the Stroke Caregiver Support System (SCSS), a supportive website designed to reduce caregiver burden	Phase I: Focus groups with stroke caregivers. Phase II: one‐on‐one structured interviews	Evaluate the usability and preliminary efficacy of the SCSS website in reducing stroke caregiver burden	Pilot usability test: 9 caregivers used the SCSS website for ~2 months. One‐group pre/post design with baseline and follow‐up assessments; included structured usability evaluation and surveys of caregiver outcomes	12‐item Zarit Burden Interview (Bédard et al. 2001); Activities of Daily Living (Katz et al. 1963); Center for Epidemiologic Studies Depression Scale (Radloff 1977); Social Support questionnaire (Barrera et al. 1981; Krause 1995; Krause and Markides 1990); Preparedness for Caregiving Scale (Archbold et al. 1990); Caregiving Self‐Efficacy scale (Steffen et al. 2002)
Chen et al. (2016)	USA	Stroke Caregiver burden and stress in caring for stroke survivors with vs. without spatial neglect	To explore factors associated with burden and stress among family caregivers of stroke survivors with spatial neglect	Semi‐structured telephone interviews	To assess caregiver burden and stress levels and related factors, comparing caregivers of stroke survivors with spatial neglect to those without spatial neglect	Telephone‐based quantitative assessments (embedded in the interviews)	Barthel Index; Life Space Questionnaire (Stalvey et al. 1999); Time Allocation Questionnaire (Blesa 2000; Clipp and Moore 1995); Caregiver Burden Scale (Elmståhl et al. 1996); Geriatric Depression Scale (Yesavage et al. 1983); Post Traumatic Stress Disorder Checklist (Weathers et al. 1994); Hopkins Verbal Learning Test (Benedict et al. 1998)
Ekstam et al. (2015)	Sweden	This study addresses the context of informal caregiving for stroke survivors, focusing on how caregivers and stroke survivors jointly experience and perceive rehabilitation needs and daily life challenges during the long‐term recovery phase	To explore the personal experience of everyday life changes among stroke survivors and their caregivers and their strategies for handling these changes 1 year after stroke	Structured open‐ended interviews	To assess the relationships between the dyad's perceptions of the stroke survivor's rehabilitation needs and stroke severity, personal characteristics, rehabilitation service use, informal caregiving, and caregiver burden	Questionnaire	Barthel Index (Mahoney and Barthel 1965); a Ware‐based rehabilitation needs questionnaire (Ware et al. 1983); the 13‐item Sense of Coherence scale (SOC‐13) (Antonovsky 1993), the Caregiver Burden Scale (Elmståhl et al. 1996)
Eriksson et al. (2021)	Uganda	Family caregivers of stroke survivors who underwent a telerehabilitation program (including caregiver counselling), life situation, perceived burden, and life satisfaction levels	To explore and describe the life situation of family caregivers to stroke survivors who received a mobile phone‐supported rehabilitation program	Semi‐structured interviews	To investigate caregivers' perceived burden and life satisfaction levels	Questionnaire	Caregiver Burden Scale (Elmståhl et al. 1996); Life Satisfaction Checklist (LiSat‐11) (Fugl‐Meyer et al. 2002)
Jordan et al. (2022)	USA	Intervention feasibility and acceptability for stroke caregivers in the U.S. Veterans Health System	To explore caregiver perceptions about the intervention's acceptability, helpfulness, and value	Semi‐structured interviews	To examine changes in depression, burden, problem‐solving, and quality of life pre−/post‐intervention	Surveys	Center for Epidemiologic Studies Depression Scale (Radloff 1977); Zarit Burden Interview—Short Form (Bédard et al. 2001); Social Problem Solving Inventory—Revised: Short Form (D’Zurilla and Sheedy 1991; D’Zurilla et al. 2002); Veterans Rand 12‐Item Health Survey (Ware and Keller 1994)
Han et al. (2024)	China	Family caregivers support resilience in stroke recovery, focusing on caregiving challenges, coping strategies, and influencing factors	To clarify factors that act as obstacles to building family resilience and to explore possible favourable factors (i.e., coping strategies) among family caregivers of stroke survivors'	Semi‐structured in‐depth interviews	To understand the status (level) of family resilience and explore its correlation with self‐efficacy, caregiver burden, family function, social support, and other influencing factors	Questionnaire‐based survey	Family Resilience Assessment Scale (FRAS); Self‐Efficacy for Managing Chronic Disease 6‐Item Scale; Caregiver Burden Scale; Family Functioning Scale; Social Support Rating Scale
King et al. (2010)	USA	Emotional distress, coping challenges, and caregiving burden during early post‐stroke recovery at home	To identify the types of problems stroke caregivers report and how those problems change over the first 4 months	Content analysis of caregivers' open‐ended problem descriptions collected at 10 counselling sessions	To assess the level of stress and coping effectiveness associated with specific caregiving problems, and to examine how these ratings relate to caregiver psychological outcomes	Numeric ratings of stress and coping during sessions, plus standardised self‐report questionnaires	Functional Independence Measure (Granger et al. 1986); Stress Appraisal and Coping Effectiveness Ratings (Toseland et al. 1989); Social Problem‐Solving Inventory (D’Zurilla et al. 1996); Center for Epidemiologic Studies Depression Scale (Radloff 1977); Bakas Caregiving Outcomes Scale (Bakas and Champion 1999); Profile of Mood States—Tension‐Anxiety subscale (McNair et al. 1992); Preparedness for Caregiving Scale (Archbold et al. 1990); McMaster Family Assessment Device (Epstein et al. 1983)
King et al. (2022)	USA	Family caregivers of stroke survivors participating in a cognitive behavioural therapy (problem‐solving) intervention study	To understand factors contributing to reduced depressive symptoms in stroke caregiver treatment responders vs. non responders	Semi‐structured telephone interviews	To assess changes in depression among stroke caregivers receiving a cognitive behavioural therapy CBT‐based problem‐solving intervention	Self‐report survey	CES‐D (Center for Epidemiologic Studies Depression Scale; Radloff 1977)
McCarthy and Lyons (2015)	USA	Spousal caregivers of stroke survivors in community‐dwelling dyads, with a focus on how discrepancies in perceived survivor functioning influence the spouse's psychological well‐being	To explore stroke survivors' and spouses' individual experiences regarding post‐stroke functioning and how these perceptions influence spouses' mental health	A structured, face‐to‐face interview	To examine the relationship between incongruence in survivor‐spouse perceptions of survivor functioning and spouse depressive symptoms	Surveys	Stroke Impact Scale (Lai et al. 2003): The Patient Health Questionnaire‐9 (PHQ‐9) (de Man‐van Ginkel et al. 2012)
McCarthy et al. (2016)	USA	Smoking‐related attitudes and behaviours within stroke survivor–caregiver dyads	To investigate the experiences of stroke survivor–caregiver couples who both smoke, regarding their smoking attitudes and behaviours	In‐depth interviews	To characterise smoking behaviour, dependence, and readiness to quit in stroke survivor caregiver dyads, and to compare survivor vs. partner measures	Structured questionnaires	Stroke Impact Scale version 3 (SIS; Lai et al. 2003); Smoking History Questionnaire (Rohsenow et al. 2013); Attitudes Toward Smoking Scale‐18 (Etter et al. 2000); Contemplation Ladder (Biener and Abrams 1991); Smoking Abstinence Self‐Efficacy Questionnaire (SASEQ; Spek et al. 2012)
Na'imah et al. (2023)	Indonesia	Caring burden among family caregivers of stroke patients in hospitals	To describe the burden experienced by family caregivers in caring for stroke patients	Semi‐structured interviews	To determine the level of burden experienced by family caregivers and examine relationships with caregiver and patient characteristics	Structured survey	Zarit Burden Interview questionnaire (Bédard et al. 2001)
Parkinson et al. (2022)	UK	Care partnerships (e.g., spouses, caregivers) experiencing anxiety and depression symptoms post‐stroke, using an online mindfulness intervention	To explore experiences and views of stroke survivors and their care partners using online Mindfulness‐based Intervention (MBI)	Semi‐structured, in‐depth interviews	To investigate clinical effectiveness and outcomes of online mindfulness‐based intervention (MBI) in reducing anxiety, depression, and improving mindfulness and mutuality among stroke survivors and care partners	Pre/post‐test within‐subject case study design	Hospital Anxiety Depression Scale (HADS; Zigmond and Snaith 1983); Mutuality Scale (MS; Archbold et al. 1990); Mindful Attention Awareness Scale (MAAS; Brown and Ryan 2003)
Rahman et al. (2024)	Malaysia	Coping with caregiving burden in a community‐based stroke rehab context	To explore coping strategies among family caregivers of stroke survivors	Semi‐structured interviews	To determine the coping strategies employed by stroke caregivers	Structured survey	Coping Strategies Inventory (Tobin et al. 1984)
Viscogliosi et al. (2019)	Canada	Family caregivers' role in supporting stroke survivors in relation to their caregiving burden	To examine the types of help caregivers provide to support older stroke survivors' participation	Semi‐structured interviews at 1, 3, and 6 months	To determine how these types of help varied according to the caregiver's burden level (different burden dimensions)	Structured questionnaire at same timepoints	Caregivers' Perceived Burden Scale (Dumont et al. 1998). Cognitive status tests (e.g., WMS‐III Logical Memory, MVPT‐V, Stroop), and social participation with the Assessment of Life Habits (LIFE‐H 3.1)
Ye et al. (2024)	China	Development and influencing factors of family resilience during stroke recovery	To understand the adaptation process of family resilience among stroke survivors and their caregivers	Semi‐structured, in‐depth interviews	To identify and examine direct and indirect relationships among family resilience, perceived social support, self‐efficacy, and self‐perceived burden	Surveys	Perceived Social Support Scale (Jiang 2001); Stroke Self‐Efficacy Questionnaire (Li et al. 2015), Self‐Perceived Burden Scale (Wu and Jiang 2010), Family Hardiness Index (Liu et al. 2014)
Yousaf et al. (2019)	Pakistan	Caregiver needs, challenges, and stressors	To explore the needs and caregiving experiences of high‐risk caregivers of stroke patients	In‐depth interviews	To identify high‐risk caregivers and assess the association between their risk status and caregiving burden or related factors	Structured questionnaire	Structured pre‐tested questionnaire (Caregiver Risk Screen Tool) used to classify caregivers by risk level

*Note:* Full references for standardised instruments listed in the “Quantitative scales used” column are provided below.

#### 5.3.1 Caregiving Topics

The included studies covered a broad range of caregiving topics, which reflect complex experiences of family caregivers and stroke survivors. Several studies explored caregiving topics such as burden, stress, and associated emotional or psychological outcomes (Chen et al. 2016; Eriksson et al. 2021; King et al. 2010; Na'imah et al. 2023). Other articles focused on coping strategies and resilience and how caregivers adapt to the burdens and responsibilities of stroke survivors (Han et al. 2024; Rahman et al. 2024; Ye et al. 2024). There are studies assessing the feasibility, acceptability, or outcomes of structured intervention programs, such as problem‐solving interventions, cognitive behavioural therapy, and web‐based support tools (Cameron et al. 2015; Caunca et al. 2020; King et al. 2022; Jordan et al. 2022). Additional studies addressed specific caregiving dynamics, including dyadic relationships, mutuality, and spousal perceptions of post‐stroke functioning (Ekstam et al. 2015; McCarthy and Lyons 2015; Viscogliosi et al. 2019). Unique topics included caregiver needs and risk identification (Yousaf et al. 2019), lifestyle and health behaviours such as smoking (McCarthy et al. 2016), and mindfulness‐based approaches for caregiver well‐being (Parkinson et al. 2022). Across the studies, caregivers were consistently positioned as key partners in recovery, either as the focus of psychosocial inquiry or as recipients of intervention and support.

#### 5.3.2 Purposes

The purposes of the included studies can be grouped into descriptive/explanatory and intervention‐focused categories. Twelve studies had descriptive or explanatory aims using mixed methods to both quantify and explore caregiving experiences. Yousaf et al. (2019) examined high‐risk caregivers by combining quantitative risk assessments with qualitative exploration of caregiving challenges. Na'imah et al. (2023) measured caregiver burden and enriched the results with semi‐structured interviews to get a full understanding of the experiences of family caregivers. Rahman et al. (2024) and Ye et al. (2024) investigated coping and family resilience by integrating quantitative measures (e.g., coping strategies, social support) with qualitative insights on adaptation processes. In the same way, studies by Chen et al. (2016), Eriksson et al. (2021), Han et al. (2024), and King et al. (2010) paired standardised measures of burden, stress, or resilience with narratives to provide a fuller understanding of caregivers' emotional and practical challenges. Other descriptive studies focused on dyadic perspectives and caregiving roles, such as the study by McCarthy and Lyons (2015), which examined how discrepancies in survivor–spouse perceptions influenced caregiver mental health, and Viscogliosi et al. (2019), which explored how caregivers support older stroke survivors' participation. Ekstam et al. (2015) studied how caregivers and stroke survivors jointly experienced rehabilitation needs and daily life challenges over time. Finally, McCarthy et al. (2016) investigated smoking behaviours and readiness to quit among caregiver–survivor dyads.

Five studies had intervention‐focused purposes and evaluated both the impact and acceptability of caregiver programs by integrating quantitative outcomes with participants' feedback. Jordan et al. (2022) and King et al. (2022) assessed depression and burden outcomes alongside qualitative feedback to understand the perceived value of problem‐solving interventions. In more detail, Jordan et al. (2022) aimed to evaluate the feasibility, acceptability, and outcomes of a psychological educational program for caregivers. The study by King et al. (2022) aimed to examine factors contributing to differences in outcomes among caregivers after receiving a cognitive behavioural problem‐solving intervention program. Caunca et al. (2020) combined usability testing with caregiver feedback to develop and refine a web‐based support system platform aimed at reducing caregivers' burden. Cameron et al. (2015) tested the feasibility and preliminary effects of the Timing It Right family support program on caregiver outcomes by merging caregiver‐reported outcomes with interviews about program experiences. Additionally, Parkinson et al. (2022) evaluated an online mindfulness intervention for stroke survivor–caregiver dyads by linking outcome changes with in‐depth participant experiences and perceived mental health benefits.

#### 5.3.3 Qualitative and Quantitative Data Used Across Studies

Across the included studies, researchers employed a range of qualitative and quantitative data collection methods to examine family caregiver experiences. The most commonly used qualitative approach was semi‐structured individual interviews. Some qualitative strands used structured open‐ended interviews or focus groups (Caunca et al. 2020; Ekstam et al. 2015; King et al. 2010). The data in the quantitative parts were mainly gathered through self‐report questionnaires, surveys, or standardised assessments. Frequently used instruments included the Zarit Burden Interview (Bédard et al. 2001) and the Caregiver Burden Scale (Elmståhl et al. 1996) to assess caregiver burden. The Center for Epidemiologic Studies Depression Scale (CES‐D) (Radloff 1977) and the PHQ‐9 (van de Man‐Ginkel et al. 2012) were used to evaluate depressive symptoms. Tools for assessing coping include the Coping Strategies Inventory (Tobin et al. 1984). Resilience was assessed using the Family Resilience Assessment Scale, and for quality of life, the Veterans RAND 12‐Item Health Survey was used (Ware and Keller 1994). Several studies combined these psychological scales with cognitive or functional assessments such as the Barthel Index (Mahoney and Barthel 1965), Life Space Questionnaire (Stalvey et al. 1999), or Assessment of Life Habits (e.g., Chen et al. 2016; Viscogliosi et al. 2019). See Table 3 for more details. See Appendix S2 for the complete list of references for the measurement scales used in the quantitative components of the included studies.

### 5.4 Methodological Features of Mixed Methods Studies

**FIGURE 2:**
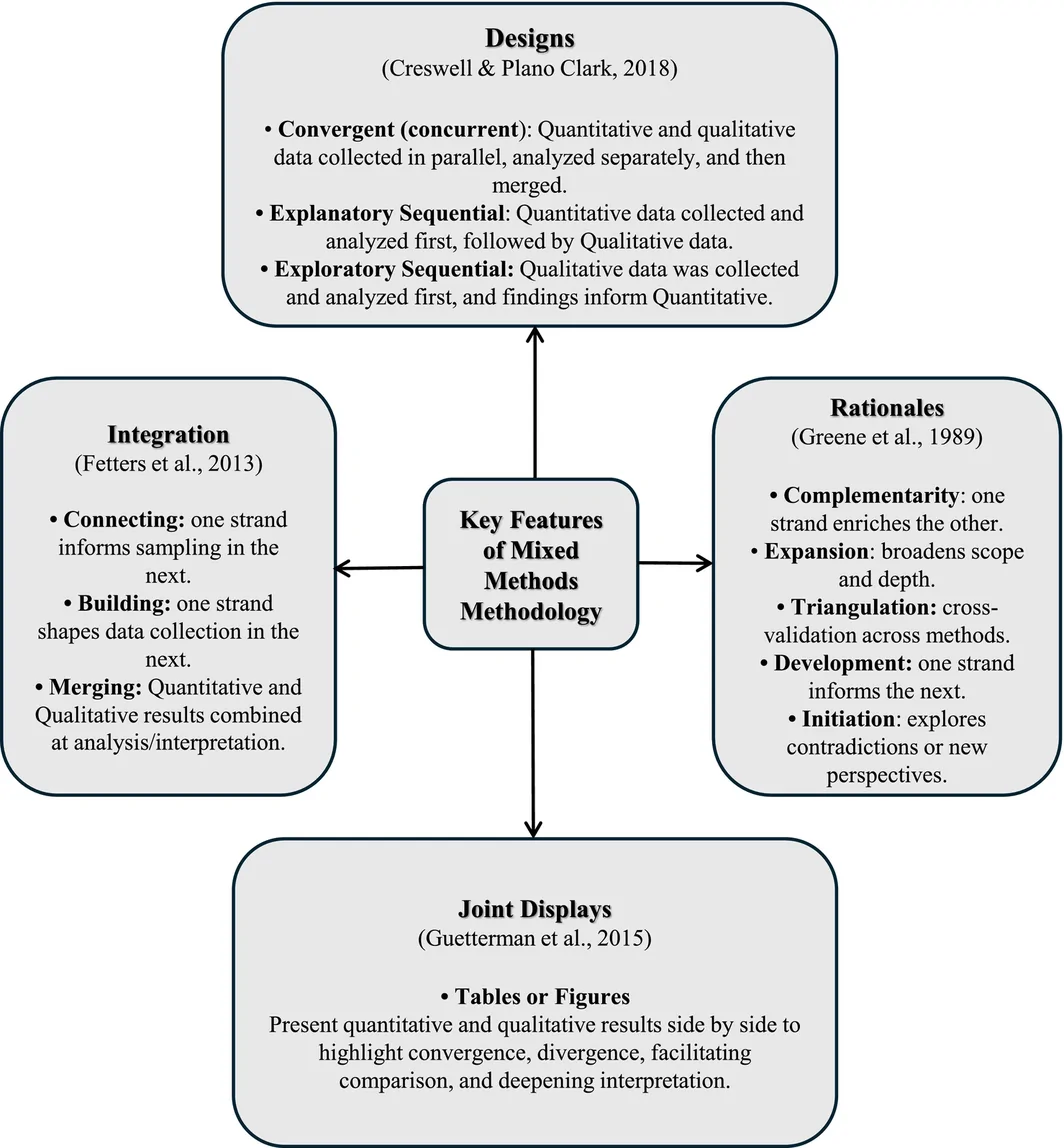
Presentation of key features of mixed method research.

This section presents the key components of applying mixed method research, including study designs, rationales for using mixed method research, integration procedures, and using joint displays across the included studies (see Table 4 and Figure 2 for a presentation of key features of mixed methods research).

**TABLE 4:** Methodological features of mixed methods studies.

Author/year	Mixed method aims/questions	MMR design (Creswell and Plano Clark 2018)		Rationale for MMR (Greene et al. 1989)		Types of integration (Fetters et al. 2013)		Joint display table
		Author statement	Reviewer interpretation	Author statement	Reviewer interpretation	Author statement	Reviewer interpretation	
Cameron et al. (2015)	To conduct a preliminary evaluation of a stroke caregiver support program's feasibility and to explore potential effects by integrating trial outcomes with caregiver interview insights	Explanatory mixed methodrandomized controlled trial	Mixed methods RCT pilot experimental design that applied an explanatory sequential core design within the RCT pilot. Mixed methods experimental design since it embeds qualitative data within a pilot randomised controlled trial to better understand intervention effects. Explanatory sequential mixed methods design as a core design, because quantitative data from a randomised controlled trial were collected first, followed by qualitative interviews to explain the findings	The study aimed to collect both quantitative and qualitative data to fully assess the program, characterise intervention delivery, and gather pilot data beyond numeric outcomes. The qualitative data helps to expand and explain the quantitative findings	1. Complementarity: qualitative interviews provided context and explanation for the quantitative outcomes (caregivers' needs, preferences, satisfaction that numbers alone could not capture). 2. Expansion: capturing aspects like intervention experience and process not measured quantitatively	Quantitative results (e.g., retention rates, outcome changes in perceived support and mastery) were considered alongside qualitative findings (interview themes about complex needs and satisfaction) in drawing conclusions about feasibility and benefit. The qualitative data highlighted how caregiver needs and preferences influenced satisfaction, complementing the modest quantitative improvements	Merging integration: in discussion and results, trial outcome data and interview themes were woven together to paint a comprehensive picture (e.g., explaining why certain outcomes did or did not change in light of caregiver feedback)	No
Caunca et al. (2020)	To develop and evaluate a stroke caregiver support intervention by integrating caregiver feedback (qualitative) with usability/outcome data (quantitative)	Three‐phase mixed methods study using an iterative, user‐centered design approach	Exploratory sequential design. The researchers collected and analysed qualitative data in the initial phases to guide the development of the caregiver support website, followed by quantitative usability testing. It also aligns with a mixed methods program evaluation, a hybrid design that integrates both data types to inform and improve intervention development	Findings from the phases were used to inform the development and refinement of the Stroke Caregiver Support System (SCSS)	1. Development: The authors used qualitative data to shape and refine the intervention (SCSS), a clear example of using one method to inform the other. 2. Complementarity: Qualitative insights added context and explanation to quantitative usability scores, enriching the understanding of user experience	The authors used qualitative findings from Phases I and II (focus groups and cognitive interviews) to guide the design and development of the caregiver support website. These were followed by Phase III usability testing, where quantitative data (e.g., SUS scores) were used to evaluate the website	Building integration at the methods level, where findings from one data type (qualitative) informed the data collection of the other (quantitative)	No
Chen et al. (2016)	To explore caregiver burden and stress in the context of spatial neglect after stroke and to identify measurable differences between caregiver groups. In addition to capturing caregivers' personal experiences and the challenges they face	Exploratory mixed method study	Convergent Mixed Methods Design: qualitative and quantitative data were collected and analysed during the same phase of the research process	To enhance the breadth and richness of the findings	1. Expansion: to extend the scope and deepen understanding of the topic, as qualitative data explored additional dimensions like economic strain and life changes not fully captured by scales. 2. Complementarity: Qualitative data provided contextual depth and illustrative examples to complement quantitative results on caregiver burden/stress	Results from both methods were compared and discussed together (e.g., quantitative group differences were reported alongside qualitative themes)	Merging in the results and discussion: The quantitative and qualitative results were interpreted in relation to each other to draw overall conclusions	No
Ekstam et al. (2015)	To examine how stroke survivors and their caregivers perceive rehabilitation needs and how these perceptions relate to stroke severity, personal and care‐related factors, while also exploring their experiences and coping strategies 1 year post‐stroke	A mixed‐methods design	Convergent (concurrent) design, questionnaires and open‐ended answers collected at the same 12‐month visit; analyses compared and combined	In the introduction, the authors stated that mixed methods offer opportunities to shed new light on the complex rehabilitation process. In the methods, they noted that qualitative findings were used to better describe and understand quantitative results. In the discussion, they emphasised that combining data provided a more comprehensive picture	1. Complementarity: to gain a more complete understanding of post‐stroke rehabilitation by clarifying and elaborating on quantitative findings. 2. Expansion: to broaden the depth and scope of insights by incorporating qualitative perspectives	In the results, the authors compared qualitative and quantitative findings using mixed model analysis under shared themes. Qualitative data helped explain the quantitative results. In the discussion, they reflected on how both data types aligned or diverged to interpret caregiver burden	Merging is evidenced in both the results and discussion, where qualitative data is used to explain and enrich quantitative findings on caregiver burden	No
Eriksson et al. (2021)	To comprehensively assess Ugandan stroke caregivers' situation by measuring burden/life satisfaction and exploring their lived experiences	A cross‐sectional mixed method design	Convergent design: Qualitative interviews and quantitative questionnaires were administered to the same individuals in one study session, and the results were interpreted together	The qualitative and quantitative data complemented each other and helped to gain a deep understanding of the caregiver family	1. Complementarity: qualitative narratives helped explain and contextualise the quantified high burden and low life satisfaction. 2. Triangulation: comparing self‐rated burden/life satisfaction with interview findings validated that caregivers who reported heavy burden also described significant challenges (cross‐confirmation of distress)	Quantitative findings showed caregivers had relatively high burden scores and low life satisfaction. These results were presented alongside qualitative. The discussion linked how the themes (heavy workload, financial strain) corresponded to the low satisfaction and high burden ratings	Merging integration: in the discussion, numerical results and qualitative themes were directly related to each other (e.g., explaining how financial difficulties reported in interviews align with low satisfaction in the financial domain)	No
Han et al. (2024)	To assess the level of family resilience among stroke survivors in China, identify its influencing factors, and establish coping strategies for family caregivers	This mixed‐methods research employed a sequential explanatory design	Explanatory sequential design (quantitative phase followed by qualitative phase to explain and expand upon the quantitative findings)	1. The qualitative phase complemented and validated the quantitative data. 2. Mixed methods were used to expand and strengthen the understanding of family resilience in this context	1. Complementarity: qualitative findings elaborate on and clarify quantitative results. 2. Triangulation: cross‐validation of findings via two strands. 3. Expansion: extending the scope and depth of understanding by adding qualitative insights to quantitative results	1. Sample for the qualitative phase was selected based on quantitative results (e.g., family resilience scores and demographics). 2. Authors integrated quantitative and qualitative results in the final stage for comprehensive interpretation	1. Connecting: Quantitative findings (family resilience scores, demographics) informed sampling and interview planning for qualitative phase. 2. Merging: Authors combined findings from both strands during interpretation to form a fuller understanding of family resilience	No
Jordan et al. (2022)	To assess the feasibility and acceptability of a 4‐week web‐based problem‐solving intervention for stroke family caregivers	Embedded mixed‐methods approach	Mixed methods experimental design that used a convergent core design within the experimental framework	The authors stated that quantitative data alone were “too deterministic and closed‐ended” and that qualitative interviews allowed for “unanticipated findings and feedback.” They also noted that a single data set was not sufficient to examine the intervention process, and that integrating both sources helped understand how participants responded	Complementarity: qualitative data were used to enhance and deepen the understanding of quantitative results	In the discussion, the authors explicitly compared quantitative and qualitative findings. They noted contradictions (e.g., no change in problem‐solving scores vs. rich qualitative use of problem‐solving) and similarities (e.g., both strands showing helpfulness of sessions)	Merging Integration: quantitative and qualitative findings were compared side‐by‐side in the discussion to interpret contradictions and identify convergence across themes	No
King et al. (2010)	To describe the problems experienced by stroke caregivers early in the caregiving role and examine how perceived stress and coping effectiveness relate to psychological outcomes	Longitudinal, mixed methods descriptive design	Convergent Design: both qualitative and quantitative data were collected in parallel, during the same timeframe. The data were analysed separately and then merged during interpretation	The authors mentioned that the combination of both data may provide a more complete picture of the caregiving experience	Complementarity: The quantitative ratings were used to enhance and clarify the meaning and significance of the caregiver‐reported problems. Expansion: The integration of numeric and narrative data allowed the researchers to extend their understanding	In the results (Table S1 and Table 5), the authors present qualitative themes (caregiving problem categories) alongside average stress and coping ratings, clearly integrating both data types for interpretation. In the discussion, they further integrate both strands by interpreting how caregivers' perceived problems relate to their psychological outcomes	Merging: The qualitative (problem themes) and quantitative (ratings and scale results) data were merged in the results and discussion	No
King et al. (2022)	To understand why some stroke caregivers responded to the depression intervention while others did not, by comparing outcomes and coping strategies	A mixed‐methods explanatory sequential design	Explanatory Sequential Design: Quantitative data were collected first through depression scores measured at multiple timepoints during the intervention. Based on these scores, participants were categorised as treatment responders or non‐responders	In the introduction, the authors report that qualitative methods were used to increase understanding of these quantitative outcomes	Complementarity: Qualitative interviews were used to enrich interpretation by adding depth and meaning to quantitative outcome differences	Caregivers were selected for interviews based on their classification as responders or non‐responders; qualitative data supplemented quantitative findings	1. Connecting integration (qualitative sampling guided by quantitative classification). 2. Merging integration, as qualitative themes were compared side‐by‐side with outcome groups to generate integrated insights	No
McCarthy and Lyons (2015)	To examine both survivor and spouse caregivers' perceptions of stroke survivor functioning and explore how incongruence affects spousal mental health using both survey data and qualitative interviews	A mixed‐methods approach	Convergent Design: Quantitative and qualitative data were collected separately but analysed and interpreted together, then merged to draw integrated inferences	Qualitative data enhanced understanding about the nuances of stroke recovery and psychological well‐being in couples	Complementarity: qualitative results were used to enrich and elaborate on quantitative associations	The authors merged findings in the discussion, where they relate statistical findings (e.g., higher incongruence linked to depressive symptoms) to qualitative themes	Merging: qualitative themes were discussed alongside quantitative findings and used to jointly interpret the impact of perception incongruence	No
McCarthy et al. (2016)	To study a health behaviour issue (smoking) in stroke caregiving couples by quantifying habits and exploring shared experiences	Embedded mixed methods design	Convergent design: quantitative surveys of smoking behaviour and qualitative dyadic interviews were conducted in the same timeframe and interpreted together	The authors explicitly identify the design as embedded mixed methods, and the stated purpose is to use quantitative data in a supportive role for qualitative data	Complementarity: quantitative results describe patterns that enhance the interpretation of dyadic experiences explored qualitatively	In discussing results, the authors considered how the qualitative themes might explain or relate to the quantitative findings (e.g., why both partners struggled to quit despite risk awareness)	Merging integration: The qualitative and quantitative results were woven together in the discussion (e.g., the theme of “conflicting feelings about individual vs. mutual concern” gives context to why readiness‐to‐quit scores were moderate). The integrated discussion leveraged both data types to suggest tailored interventions for couples	No
Na'imah et al. (2023)	To describe caregiver burden and explore associated demographic and patient‐related factors among stroke caregivers	Sequential explanatory mixed methods design	Explanatory sequential design (The quantitative phase is completed first, and the qualitative phase follows to explore or explain quantitative findings)	Not explicitly stated by the authors	No clear, explicit, or implicit rationale was provided by the authors	In the discussion section, the authors explicitly compared quantitative results (no correlation between caregiver gender and burden) with qualitative findings (female caregivers reported caregiving difficulties)	Merging Integration: In the discussion, the authors merged quantitative and qualitative findings by directly discussing quantitative data alongside qualitative narratives	No
Parkinson et al. (2022)	To evaluate an online mindfulness intervention for stroke survivor–caregiver dyads by linking outcome changes with in‐depth participant experiences	Explanatory sequential mixed methods case study design	Explanatory sequential mixed methods design (quantitative data collection and analysis completed first, informing the subsequent qualitative phase to explore and explain initial findings)	To provide a deeper understanding and explanation of quantitative results by exploring participants' lived experiences through qualitative analysis. Qualitative data was used specifically to offer an insider perspective, allowing researchers to capture the nuanced meanings and complexities of participants' experiences with the mindfulness‐based intervention	1. Complementarity, as the qualitative phase was used to elaborate on and help interpret the quantitative results. 2. Expansion, as the qualitative data provided additional insights into the lived experience of care partnerships that were not captured by the numerical data	The interpretation of findings combined quantitative outcomes with qualitative narratives to explain complex and contradictory experiences	Merging integration: The authors combined quantitative and qualitative findings during the discussion. They used descriptive statistics and clinical outcome data alongside the derived themes to construct a cohesive understanding of participants' experiences	No
Rahman et al. (2024)	To determine and explore coping strategies practiced by stroke family caregivers	Convergent parallel mixed methods research design	Convergent design involves simultaneously collecting and independently analysing quantitative and qualitative data, followed by merging analyses and results	1. MMR provides a more thorough understanding of the problem. 2. MMR is a way to deliver a wide‐ranging point of view. 3. MMR allows for comparing quantitative and qualitative data to reflect participants' viewpoints	1. Complementarity rationale. 2. Expansion. 3. Triangulation rationale, which enhances validity by cross‐verifying findings from both data sources	1. The quantitative and qualitative data were integrated using joint displays. 2. Table S1 mentions categories, subcategories, themes, and quotes from both datasets	1. Merging Integration: The joint display demonstrated in Table 3 reflects merging integration. 2. Table S1 integrates quantitative coping strategies with qualitative quotes and themes, reflecting merging	Yes Table 3
Viscogliosi et al. (2019)	To examine the relationship between the types of help caregivers provide and the different dimensions/levels of their burden	This study used a mixed method design	Convergent‐concurrent mixed‐methods design: qualitative and quantitative data were collected at the same time points and analysed together	The mixed‐method design was very useful in gaining an in‐depth understanding of the relationship between types of help and the level of different dimensions of their burden. It also shed light on the rationale for the type of help provided	1. Complementarity: combining qualitative and quantitative data provided a more comprehensive understanding of how caregiving strategies relate to burden. 2. Triangulation: Quantitative measures (e.g., cognitive tests, burden scores) enhanced validity and contextualised the qualitative findings	1. The pragmatic approach adopted in this study allowed authors to consider both the qualitative and quantitative components in analyses and interpretations. 2. Caregivers were selected for interviews using maximum variation based on quantitative characteristics like cognitive severity, care setting, and service use. 3. Data were analysed independently, then compared and merged using an analysis matrix	1. Integration occurred at the method level, as the authors designed and implemented the study using a pragmatic paradigm. 2. Connecting: Quantitative data (e.g., cognitive scores, service use) informed the purposeful sampling strategy for the qualitative strand. 3. Merging occurred during analysis, results, and discussion. The authors used a matrix (Table 3), which functions as a joint display aligning qualitative themes (types of help) with quantitative burden dimensions, allowing the authors to interpret patterns of convergence and divergence	Yes Table 3
Ye et al. (2024)	To identify consistency and discrepancies between qualitative and quantitative findings regarding family resilience	Explanatory sequential mixed methods design	Explanatory sequential (the whole Quantitative strand, including analysis, is finished, and based on the quantitative findings, the qualitative phase starts)	The authors used integration to understand family resilience better, assess complementarity between data sources, and explore consistency and discrepancies	1. Complementarity to enhance understanding by comparing different types of findings 2. Initiation to uncover and explain inconsistencies between strands	1. The interview questions for the qualitative part were shaped by the quantitative findings. 2. Joint display in Table S1 used to compare qualitative and quantitative findings for complementarity and inconsistencies	1. Building integration by developing interview questions based on the quantitative aspects of the study. 2. Merging Integration, where both quantitative and qualitative results are combined using joint display. 3. Connection integration in that the scores (above 75th % percentile) from the quantitative strand are used to inform sampling in the Qualitative strand	Yes Table S1
Yousaf et al. (2019)	To identify high‐risk caregivers and explore the challenges they face	The study was described as a “mixed‐method approach”	Explanatory Sequential Design: caregivers were first screened quantitatively to determine risk level; qualitative interviews followed with high‐risk caregivers	The authors mention using qualitative interviews to gain deeper insight into caregiver needs that were not captured through the risk screening tool	Complementarity: qualitative data enriched understanding by elaborating on the challenges experienced by those identified as high risk in the survey	1. Based on the quantitative risk screening tool, nine caregivers identified as high‐risk were selected for qualitative interviews. 2. In the results and discussion, qualitative themes were interpreted in relation to the high‐risk classification	1. Connecting Integration: The quantitative results (risk classification) guided the selection of participants for the qualitative strand. 2. Merging integration: qualitative themes were used to explain or expand quantitative findings during interpretation	No

#### 5.4.1 Mixed Methods Designs

Among the 17 included studies, convergent and explanatory sequential designs dominated the methodological landscape. Convergent designs were the most common design used (*n* = 8) (Chen et al. 2016; Ekstam et al. 2015; Eriksson et al. 2021; King et al. 2010; McCarthy and Lyons 2015; McCarthy et al. 2016; Rahman et al. 2024; Viscogliosi et al. 2019). These studies collected and analysed qualitative and quantitative data during the same timeframe and then compared and merged the results to provide a comprehensive picture of caregiving burden, coping, and well‐being. For instance, Rahman et al. (2024) collected coping strategy surveys alongside qualitative interviews and integrated both sets of results by combining coping styles with themes about experiences.

Explanatory sequential designs were employed in seven studies (Cameron et al. 2015; Han et al. 2024; King et al. 2022; Na'imah et al. 2023; Parkinson et al. 2022; Ye et al. 2024; Yousaf et al. 2019). These studies typically used quantitative results (e.g., depression scores, burden indices) to inform qualitative sampling and used qualitative results to generate in‐depth interpretations of the numerical findings. For example, King et al. (2022) classified caregivers as treatment responders or nonresponders based on depression outcomes; then qualitatively, they explored these subgroups to explain differences in coping responses.

Only one study employed an exploratory sequential design (Caunca et al. 2020), which used qualitative feedback from caregivers to design a web‐based support intervention and then quantitatively evaluated its usability and preliminary effects. One study, Jordan et al. (2022) reported that they used an embedded design, where qualitative interviews were incorporated within a primarily quantitative one‐group pretest/posttest intervention study. The qualitative component was used to explore participants' perceptions of the intervention and to support interpretation of the outcome data. This approach is aligned with Creswell and Plano Clark's (2018) hybrid design, which is a mixed methods experimental design that uses a convergent core design within the experimental framework.

Although most studies identified using mixed method research, only seven articles explicitly identified a type of mixed methods design by name. Several lacked clarity in defining or referencing their chosen design. Some studies mentioned using mixed method design without specifying the type or providing an appropriate methodological reference. For instance, Yousaf et al. (2019) referred to their study's design as a “mixed‐method approach” but did not label it as an explanatory sequential design, even though its structure aligns with that core mixed methods design.

#### 5.4.2 Rationales

Among the 17 studies, complementarity was the most common rationale, cited or implied in the majority of the included studies. Researchers frequently used qualitative data to enhance, explain, or provide deeper insight into the quantitative data. For example, Jordan et al. (2022) mentioned that quantitative data were insufficient for examining and evaluating the intervention process and integrating data from both strands provided a better understanding of participants' responses. Also, King et al. (2022) used qualitative insights to interpret differences in quantitative outcomes. Ye et al. (2024) emphasized that integrating both strands was important for achieving a comprehensive understanding of family resilience and helping to explore discrepancies between strands, reflecting both complementarity and initiation rationales.

Expansion was also observed in several studies (Cameron et al. 2015; Chen et al. 2016; Ekstam et al. 2015; Han et al. 2024), where qualitative data broadened understanding by adding new dimensions (e.g., unmet needs, caregiver perceptions) not captured by quantitative measures. Triangulation appeared in studies like Han et al. (2024), Viscogliosi et al. (2019), and Eriksson et al. (2021), where qualitative and quantitative data were used to cross‐validate findings. For example, in Han et al. (2024), qualitative interviews confirmed and elaborated on quantitative measures of family resilience, while in Viscogliosi et al. (2019), burden scores aligned with qualitative reports of strain, and in Eriksson et al. (2021), high burden and low life satisfaction scores were echoed in caregiver narratives of financial strain and heavy workload. Initiation used to uncover inconsistencies was explicitly noted in Ye et al. (2024), where qualitative findings were used to explore discrepancies in family resilience scores. Only one study adopted the development rationale (Caunca et al. 2020). In this study, the authors used qualitative caregiver feedback to shape and refine the design of a web‐based support intervention. In contrast, one study (Na'imah et al. 2023) did not clearly state or imply a rationale for using mixed method research. However, their explanatory sequential design suggests that complementarity was the intended purpose, as qualitative interviews were employed to deepen the interpretation of the burden data.

#### 5.4.3 Integration Procedures and Joint Displays

Integration across the 17 included mixed methods studies occurred primarily through merging and connecting strategies, with building integration used less frequently (Fetters et al. 2013). The connecting integration approach was evident in explanatory sequential designs, where results from the quantitative phase informed the qualitative phase sample. Yousaf et al. (2019) used caregiver risk scores from surveys to identify high‐risk participants for follow‐up interviews, and King et al. (2022) selected caregivers for qualitative analysis based on whether they were classified as intervention responders or nonresponders. Similarly, Ye et al. (2024) used quantitative scores (above the 75th percentile) to guide sampling for the qualitative strand, Han et al. (2024) drew on family resilience scores and demographic data to select samples for the qualitative phase, and Viscogliosi et al. (2019) relied on cognitive scores and service use data to inform purposeful sampling for the qualitative phase. Building integration was less common and was applied in just two studies. The first one by Ye et al. (2024), where quantitative results were used to shape qualitative interview questions. The second article demonstrated building integration by Caunca et al. (2020), as findings from the initial qualitative focus groups (Phase I) were used to refine the Stroke Caregiver Support System (SCSS) prototype and guide the content evaluated in subsequent structured interviews (Phase II). Feedback from Phase II interviews further informed modifications to modules and videos before conducting the usability testing (Phase III).

Merging was evident in several studies, for example Rahman et al. (2024) combined quantitative coping scores with qualitative themes during interpretation in the discussion section. Jordan et al. (2022) merged findings in the discussion by comparing outcome scores from the quantitative strand with interview findings to explore how caregivers perceived the intervention, noting both convergence and discrepancies. Na'imah et al. (2023) used merging at the interpretation level, discussing how qualitative accounts helped explain or contrast with the quantitative burden data. Lastly, Yousaf et al. (2019) used merging by interpreting qualitative themes in relation to the high‐risk caregiver classification identified through survey data to explain or expand quantitative findings. In addition, merging was evident in the use of joint displays in several studies, where quantitative and qualitative results were presented side by side to facilitate direct comparison and highlight convergence, divergence, or expansion of findings.

### 5.5 Joint Displays

Only three studies employed tables functioning as joint displays to present integrated findings (Rahman et al. 2024; Viscogliosi et al. 2019; Ye et al. 2024). Rahman et al. (2024) explicitly stated that the quantitative and qualitative data were integrated using joint displays. These authors used a joint display to facilitate integration by linking numerical findings with illustrative caregiver quotes, offering a clearer understanding of coping strategies. Ye et al. (2024) included a joint display table comparing quantitative resilience scores with qualitative themes. It showed alignment across family resilience domains and indicated whether findings were consistent, inconsistent, or supplementary, and this helps in providing richer insights into the development of family resilience.

While the authors in the study by Viscogliosi et al. (2019) did not explicitly label a table as a ‘joint display,’ Table 4 functioned as one by aligning qualitative categories of caregiver help with quantitative burden measures, effectively merging data strands to reveal patterns of support and perceived burden. Joint displays are considered state of the art practices in mixed method research because they provide a clear and systematic way to integrate datasets, highlight convergence or divergence, and enhance interpretive depth (Creswell and Plano Clark 2018; Fetters et al. 2013; Guetterman et al. 2015). The identification of three studies using this technique underscores its methodological rigor and reflects the innovative application of joint displays within the included literature.

## 6 Discussion

This is the first methodological review that aims to examine the ways in which mixed method research was applied within the family caregivers of stroke survivors. This paper provided an overview of the types of qualitative and quantitative data used, the caregiving topics explored, and how researchers approach mixed method key components such as rationale, design, integration, and the use of joint display. Most of the included articles were conducted in the United States, which is similar to the results of several methodological systematic reviews (Fàbregues et al. 2022; Fàbregues et al. 2023; Fàbregues et al. 2025; Granikov et al. 2020). The predominance of studies in the United States reflects the foundational role in the evolution of mixed methods research in the country, which is supported by robust methodological training and established academic frameworks (Creswell and Sinley 2017).

In terms of topic consideration, the findings demonstrate that mixed method studies among stroke family caregivers have been used to investigate a range of caregiver‐related issues, including burden, coping, resilience, mental health, caregiver‐survivor dyads, and interventions aimed to support caregivers' outcomes. These findings align with prior literature, which shows that stroke caregivers experience substantial burden and emotional distress and would benefit from targeted, structured interventions (Bakas et al. 2014; Kokorelias et al. 2020). Moreover, resilience and adaptive coping have emerged as modifiable targets that can indirectly enhance caregiving ability (Han et al. 2024; Wang et al. 2023).

In terms of data types, qualitative and quantitative methods were used across studies, but their reporting quality varied. While prior reviews, such as O'Cathain et al. (2008), identified transparency issues in the qualitative components of mixed methods health research, our review revealed the opposite trend. Based on the MMAT appraisal, all studies demonstrated stronger reporting and methodological clarity in their qualitative parts. In contrast, the quantitative parts, especially in descriptive studies, lacked essential details about sampling representativeness, measurement appropriateness, and response rate handling. Several quantitative components were rated as “Cannot tell” in the MMAT due to insufficient reporting in these areas. This may reflect challenges researchers face in designing robust quantitative methods for caregiver populations, or it may indicate a broader pattern of underreporting quantitative procedures in mixed methods caregiving studies.

Methodologically, the application of mixed methods in these studies demonstrated some similarities and inconsistencies regarding the application and reporting of mixed method research. For the design, among the included studies, convergent and explanatory sequential designs dominated the methodological landscape. Convergent designs are the most commonly used, consistent with findings from other methodological reviews in library and information science (Granikov et al. 2020). Notably, reporting on design types was often inconsistent. Several articles did not clearly identify their mixed methods design or omitted references to standard typologies, instead describing only a general “mixed methods approach”. This lack of design clarity can impede readers' understanding of how studies were conducted. A notable concern regarding reporting the design was the lack of design clarity and citation. More importantly, when authors fail to identify or define their mixed methods design, they also miss the opportunity to learn from methodological writings specific to the selected type of design and implement best practices to achieve quality integration. For example, Na'imah et al. (2023) and Ye et al. (2024) followed an explanatory sequential approach. However, they did not cite any methodological references or give any brief explanation or definition for the design. Yousaf et al. (2019) mentioned using a mixed methods approach in general but did not specify a core design or cite any relevant mixed method research literature. While some other articles mentioned an appropriate reference for their design, they did not provide any definition or explanation for their design. These results of the lack of explicit use of mixed method design types aligned with a review of mixed methods studies in health services research, which report a lack of justification for mixed method design and describe separate parts of the study (O'Cathain et al. 2008). Also, another methodological systematic review among palliative and end‐of‐life care resulted in a need for improvement in reporting the type of mixed methods design (Fàbregues et al. 2020). Another review resulted in a need for the description of the design used (Granikov et al. 2020; Fàbregues et al. 2022). Ensuring clear identification and definition of the design up front would strengthen methodological rigour and reproducibility in future mixed methods caregiving studies.

Complementarity emerged as the most frequently reported rationale for using mixed methods in the included studies. While many authors explicitly stated this rationale, several described it only implicitly or without sufficient methodological detail, and others did not discuss a rationale at all, indicating variability in how well the rationale was justified. This aligns with findings of another methodological systematic review by Fàbregues et al. (2020), who noted that many studies failed to adequately justify their use of mixed method research or specify the rationale guiding it. The frequent use of complementarity suggests that researchers value the ability to integrate qualitative and quantitative data to enrich, explain, or contextualise findings. The absence of a clear rationale for the use of mixed method design in some studies raises concerns about the actual intentionality behind the use of this approach. The clear rationale in mixed method research is essential for aligning the approach with the study's purpose and ensuring purposeful integration (Bryman 2006; Greene et al. 1989).

Data integration techniques varied across the reviewed studies, with room for improvement in integration depth and transparency. It is encouraging that integration of qualitative and quantitative strands was present in all studies, but the depth and clarity of integration varied. Most studies used merging integration, typically during interpretation, while connecting and building integration appeared in sampling and data collection. The majority of studies implemented integration at the interpretation phase, often without a clear explanation of how findings from both strands were combined, which means that integration often occurred in the written discussion of results rather than through explicit mixed methods procedures during data collection or analysis. Many authors wove qualitative themes and quantitative outcomes together in the discussion part (e.g., discussing how interview insights explained or expanded upon survey trends), but without detailing a formal integration protocol or figures to help readers understand the process of integration. While this narrative weaving achieves a basic level of integration, it sometimes leaves unclear how the data was linked. In only a minority of articles did studies demonstrate more explicit integration strategies. A key area for growth is to move beyond narrative interpretation and implement merging integration at the analysis stage, for example, through the use of joint displays that allow direct comparison of qualitative and quantitative results and make integration more transparent (Fetters et al. 2013). These results aligned with many of the methodological systematic reviews in different fields that resulted in the need for more details on the type of integration used and reporting it in a transparent way, and using data visualisation in integration (Fàbregues et al. 2020; Fàbregues et al. 2022; Zhou et al. 2024).

Only three studies employed joint displays, a recommended tool for enhancing integration transparency and coherence (Fetters et al. 2013). This can be a concern since joint displays are a good way to present integration and interpret results (Guetterman et al. 2021). There is room to improve the use of integration, especially with the use of joint displays (tables or figures). This aligned with a review that advocates the use of joint displays in visual features, including charts, graphs, maps, and images (Guetterman et al. 2021).

Several studies in this review serve as methodological exemplars that highlight best practices in the application of mixed method research. Rahman et al. (2024) clearly mentioned their mixed method design, provided multiple rationales for using mixed method research (expansion, complementarity, and triangulation), and demonstrated robust merging integration by using joint display to align coping survey results with qualitative themes. Ye et al. (2024) offered a rigorous application of explanatory sequential design, using connecting, merging, and building integration as quantitative resilience scores guided qualitative sampling and analysis, mentioned multiple rationales for using mixed method research, and further strengthened transparency through a joint display that compared consistencies and discrepancies across strands. Similarly, Viscogliosi et al. (2019) mentioned complementarity and triangulation as the rationale. Applied connecting and merging integration by using Table 4 as a joint display, thereby achieving integration at the analysis level and making explicit how qualitative caregiving categories converged with or diverged from quantitative burden measures.

### 6.1 Implications

Mixed method research provides valuable insights into family caregiving for stroke survivors by capturing both the measurable and lived experience outcomes of family caregivers. Adopting mixed method research helps researchers gain a more holistic understanding of caregiver burdens, coping mechanisms, and resilience. Combining qualitative and quantitative approaches helps researchers and healthcare professionals, including nurses, obtain a more comprehensive understanding of family caregivers' situations.

The effectiveness of using mixed method research findings in informing research, policy, and practice depends on the clarity and transparency of the methodological application of mixed method research. A clear rationale statement, explicit integration strategies, intentional mixed method design, and using joint displays either in tables or figures not only enhances methodological rigour but also enables replication and synthesis across studies. Such transparency allows health care providers, including nurses, nurse educators, and policymakers, to interpret findings with confidence and to apply them in designing evidence‐based interventions, caregiver education programs, and policy reforms.

Promoting consistent use of mixed method key components in stroke caregiving research ensures that the resulting evidence is not only scientifically robust but also practically meaningful. Integrated findings in mixed method studies can identify both the statistical effectiveness and perceived acceptability of family caregiver support programs, making them more adaptable to real‐world settings. Additionally, encouraging methodologically sound mixed method research will elevate the quality and utility of evidence, ultimately improving both caregiver well‐being and patient recovery outcomes. This enhanced methodological rigor provides healthcare providers with more trustworthy and context‐rich data to guide assessment, care planning, and discharge coordination, as a result supporting effective caregiving in practice. Lastly, healthcare organisations can develop system‐level strategies that align with caregivers' actual needs and lived experiences based on this evidence.

### 6.2 Recommendation for Future Mixed Methods Studies

1. Increase awareness and training in mixed methods research among researchers, particularly regarding design typologies and integration procedures. Researchers should be encouraged to explicitly cite methodological sources to enhance intentionality, coherence, and confidence in applying mixed method research rigorously.
2. Provide an explicit and clearly articulated rationale for selecting a mixed method research approach directly aligned with established frameworks (e.g., Greene et al. 1989) to ensure coherence between the research purpose, questions, and methods. A clear rationale for using mixed method research helps readers understand the purpose and value of integrating qualitative and quantitative strands and enhances transparency.
3. Clearly define and cite mixed method research designs to improve transparency and reproducibility. Naming the specific design (e.g., explanatory sequential) helps other researchers replicate the approach and ensures methodological clarity, which is essential for scholarly communication.
4. Systematically employ integration strategies (e.g., merging, connecting, building), clearly describe each step of integration and its relevance to generating comprehensive insights into caregiving research. Integration is the core feature of mixed method research and explaining how it is achieved supports more meaningful synthesis and interpretation of findings.
5. Incorporate joint displays to present integration and interpret results. Joint displays improve understanding of how qualitative and quantitative findings relate and make integration more transparent, supporting richer insights for researchers, practitioners, and policymakers.
6. Editors and reviewers should encourage authors to report mixed method research components in detail, including a clearly stated rationale and design, and provide detailed evidence of data integration (e.g., through joint displays or explicit linking of data sources) with appropriate references. Strengthening peer‐review standards will promote more rigorous and consistent reporting practices across published mixed methods studies.
7. Encourage and support the publication of methodologically robust exemplars specifically within the stroke caregiving research context, serving as practical references to strengthen methodological quality and rigour in the field. Exemplars guide future researchers by modelling best practices and demonstrating how high‐quality mixed method research can advance both science and care.

### 6.3 Strengths and Limitations

The systematic methodological focus and explicit examination of the application of mixed method research in stroke caregiving research are one of the main strengths in this review. This approach provides valuable insights into how methodological rigour can enhance caregiving research and help the family caregiver population. Additionally, adopting well‐established frameworks when doing mixed method research (Creswell and Plano Clark 2018; Fetters et al. 2013; Greene et al. 1989) supports consistency and reproducibility. Unlike content‐driven reviews, our approach sheds light on the research process itself, offering guidance that can elevate the quality of future studies in this field. However, this review has some limitations. Our selection criteria focused on English‐language, peer‐reviewed articles, which may have led to the exclusion of important studies published in other languages. Our review is based only on the information contained within article texts, which cannot represent all of the considerations undertaken by authors. Additionally, inconsistencies in reporting designs and integration procedures among the included studies limit deeper methodological comparisons.

## 7 Conclusion

This review examined the methodological application of using mixed method research in studies of family caregiving for stroke survivors. The results showed that while mixed method research is being used to address diverse family caregiving topics, the methodological application of it varies in clarity and depth. This variation reflects the evolving nature of mixed method research in family caregiving research and highlights the need for more precise application of key components of mixed method research. The review offers insights for researchers aiming to apply mixed method research in family caregiving studies and encourages greater methodological consistency and clarity to enhance the rigour of future work. Strengthening the application and reporting of mixed method research in stroke caregiving not only advances scientific rigour but also contributes directly to more effective, caregiver‐centered clinical interventions and policy innovations. It may also inform mixed method research methodologists about how mixed method research is currently being utilised in family caregiving and where further development is needed.

## Funding

The authors have nothing to report.

## Conflicts of Interest

The authors declare no conflicts of interest.

## Supporting information


**Appendix S1:** PRISMA_2020_checklist.

**Appendix S2:** Instruments references.

## Data Availability

Data sharing not applicable to this article as no datasets were generated or analysed during the current study.
