# Supplementary material for: Mixed Methods Research on Family Caregiving for Stroke Survivors: A Methodological Systematic Review
Source: J Adv Nurs. 2026 Mar 4;82(10):8891–920. doi: 10.1111/jan.70534 (PMC13576757; doi:10.1111/jan.70534)
Supplement: Supplementary file 2 [file JAN-82-8891-s002.docx]

**Appendix B**
*References for Standardized Instruments Used in Included Studies*

Table S1 Caregiving topics and study purposes.

| Author/year | Country | Caregiving Topics | Qualitative strand | | Quantitative strand | | |
| --- | --- | --- | --- | --- | --- | --- | --- |
|  |  |  | Qualitative aim/question | Qualitative method used | Quantitative aim/question | Quantitative method used | Quantitative scales used |
| Cameron et al. (2015) | Canada | Family/friend caregivers of first-time stroke survivors across acute and community care, participating in a phased support intervention program | To gather family caregivers' experiences and satisfaction with a new stroke family support program (Timing It Right) | In-depth qualitative interviews and qualitative data from the recruitment and intervention delivery records | To pilot-test the feasibility and preliminary effects of the Timing It Right Stroke Family Support Program on caregiver outcomes | Multi-site randomised controlled trial (pilot) | 20-item Medical Outcomes Study Social Support Scale (Sherbourne & Stewart, 1991): Positive Affect  Scale (Watson et al. 1988); 20-item Centre for Epidemiological Studies Depression Scale (CESD) (Radloff 1977); Pearlin's  7-item Mastery Scale (Cameron et al. 2015); Stroke Knowledge Test (Sullivan & Dunton, 2004); Caregiving Impact scale and Caregiving Assistance Scale (Cameron et al. 2002; Sullivan & Dunton, 2004) |
| Caunca et al. (2020) | USA | Development of a web-based support system for family caregivers aimed at reducing caregiver burden | To identify the needs, concerns, and feedback of stroke caregivers to guide the development of the Stroke Caregiver Support System (SCSS), a supportive website designed to reduce caregiver burden | Phase I: Focus groups with stroke caregivers.  Phase II: one-on-one structured interviews | Evaluate the usability and preliminary efficacy of the SCSS website in reducing stroke caregiver burden | Pilot usability test: 9 caregivers used the SCSS website for ~2 months. One-group pre/post design with baseline and follow-up assessments; included structured usability evaluation and surveys of caregiver outcomes | 12-item Zarit Burden Interview (Bédard et al. 2001); Activities of Daily Living (Katz et al. 1963); Center for Epidemiologic Studies Depression Scale (Radloff 1977); Social Support questionnaire (Barrera et al. 1981; Krause, 1995; Krause&Markides, 1990); Preparedness for Caregiving Scale (Archbold et al. 1990); Caregiving Self-Efficacy scale (Steffen et al. 2002) |
| Chen et al. (2016) | USA | Stroke Caregiver burden and stress in caring for stroke survivors with vs. without spatial neglect | To explore factors associated with burden and stress among family caregivers of stroke survivors with spatial neglect | Semi-structured telephone interviews | To assess caregiver burden and stress levels and related factors, comparing caregivers of stroke survivors with spatial neglect to those without spatial neglect | Telephone-based quantitative assessments (embedded in the interviews) | Barthel Index; Life Space Questionnaire (Stalvey et al.1999); Time Allocation Questionnaire (Blesa, 2000; Clipp & Moore, 1995); Caregiver Burden Scale (Elmståhl et al. 1996); Geriatric Depression Scale (Yesavage et al. 1983); Post Traumatic Stress Disorder Checklist (Weathers et al. 1994); Hopkins Verbal Learning Test (Benedict et al.1998) |
| Ekstam et al. (2015) | Sweden | This study addresses the context of informal caregiving for stroke survivors, focusing on how caregivers and stroke survivors jointly experience and perceive rehabilitation needs and daily life challenges during the long-term recovery phase | To explore the personal experience of everyday life changes among stroke survivors and their caregivers and their strategies for handling these changes 1 year after stroke | Structured open-ended interviews | To assess the relationships between the dyad's perceptions of the stroke survivor's rehabilitation needs and stroke severity, personal characteristics, rehabilitation service use, informal caregiving, and caregiver burden | Questionnaire | Barthel Index (Mahoney and Barthel 1965); a Ware-based rehabilitation needs questionnaire (Ware et al. 1983); the 13-item Sense of Coherence scale (SOC-13) (Antonovsky, 1993), the Caregiver Burden Scale (Elmståhl et al. 1996) |
| Eriksson et al. (2021) | Uganda | Family caregivers of stroke survivors who underwent a telerehabilitation program (including caregiver counselling), life situation, perceived burden, and life satisfaction levels | To explore and describe the life situation of family caregivers to stroke survivors who received a mobile phone-supported rehabilitation program | Semi-structured interviews | To investigate caregivers' perceived burden and life satisfaction levels | Questionnaire | Caregiver Burden Scale (Elmståhl et al. 1996); Life Satisfaction Checklist (LiSat-11) (Fugl-Meyer et al. 2002) |
| Jordan et al. (2022) | USA | Intervention feasibility and acceptability for stroke caregivers in the U.S. Veterans Health System | To explore caregiver perceptions about the intervention's acceptability, helpfulness, and value | Semi-structured interviews | To examine changes in depression, burden, problem-solving, and quality of life pre−/post-intervention | Surveys | Center for Epidemiologic Studies Depression Scale (Radloff 1977);  Zarit Burden Interview—Short Form (Bédard et al. 2001); Social Problem Solving Inventory—Revised: Short Form (D'Zurilla & Sheedy, 1991; D'Zurilla et al. 2002);  Veterans Rand 12-Item Health Survey (Ware and Keller 1994) |
| Han et al. (2024) | China | Family caregivers support resilience in stroke recovery, focusing on caregiving challenges, coping strategies, and influencing factors | To clarify factors that act as obstacles to building family resilience and to explore possible favourable factors (i.e., coping strategies) among family caregivers of stroke survivors' | Semi-structured in-depth interviews | To understand the status (level) of family resilience and explore its correlation with self-efficacy, caregiver burden, family function, social support, and other influencing factors | Questionnaire-based survey | Family Resilience Assessment Scale (FRAS); Self-Efficacy for Managing Chronic Disease 6-Item Scale; Caregiver Burden Scale; Family Functioning Scale; Social Support Rating Scale |
| King et al. (2010) | USA | Emotional distress, coping challenges, and caregiving burden during early post-stroke recovery at home | To identify the types of problems stroke caregivers report and how those problems change over the first 4 months | Content analysis of caregivers' open-ended problem descriptions collected at 10 counselling sessions | To assess the level of stress and coping effectiveness associated with specific caregiving problems, and to examine how these ratings relate to caregiver psychological outcomes | Numeric ratings of stress and coping during sessions, plus standardised self-report questionnaires | Functional Independence Measure (Granger, Hamilton, & Sherwin, 1986); Stress Appraisal and Coping Effectiveness Ratings (Toseland, Rossiter, & Labrecque, 1989); Social Problem-Solving Inventory (D'Zurilla, Nezu, & Maydeu-Olivares, 1996); Center for Epidemiologic Studies Depression Scale (Radloff 1977); Bakas Caregiving Outcomes Scale (Bakas & Champion, 1999); Profile of Mood States—Tension-Anxiety subscale (McNair, Lorr, & Droppleman, 1992); Preparedness for Caregiving Scale (Archbold, Stewart, Greenlick, & Harvath, 1990); McMaster Family Assessment Device (Epstein, Baldwin, & Bishop, 1983) |
| King et al. (2022) | USA | Family caregivers of stroke survivors participating in a cognitive behavioural therapy (problem-solving) intervention study | To understand factors contributing to reduced depressive symptoms in stroke caregiver treatment responders vs. non responders | Semi-structured telephone interviews | To assess changes in depression among stroke caregivers receiving a cognitive behavioural therapy CBT-based problem-solving intervention | Self-report survey | CES-D (Center for Epidemiologic Studies Depression Scale; Radloff 1977) |
| McCarthy and Lyons (2015) | USA | Spousal caregivers of stroke survivors in community-dwelling dyads, with a focus on how discrepancies in perceived survivor functioning influence the spouse's psychological well-being | To explore stroke survivors' and spouses' individual experiences regarding post-stroke functioning and how these perceptions influence spouses' mental health | A structured, face-to-face interview | To examine the relationship between incongruence in survivor-spouse perceptions of survivor functioning and spouse depressive symptoms | Surveys | Stroke Impact Scale (Lai et al. 2003): The Patient Health Questionnaire-9 (PHQ-9) (van de Man-Ginkel et al. 2012) |
| McCarthy et al. (2016) | USA | Smoking-related attitudes and behaviours within stroke survivor–caregiver dyads | To investigate the experiences of stroke survivor–caregiver couples who both smoke, regarding their smoking attitudes and behaviours | In-depth interviews | To characterise smoking behaviour, dependence, and readiness to quit in stroke survivor caregiver dyads, and to compare survivor vs. partner measures | Structured questionnaires | Stroke Impact Scale version 3 (SIS; Lai et al. 2003); Smoking History Questionnaire (Rohsenow et al. 2013); Attitudes Toward Smoking Scale-18 (Etter et al. 2000); Contemplation Ladder (Biener & Abrams, 1991); Smoking Abstinence Self-Efficacy Questionnaire (SASEQ; Spek et al. 2012) |
| Na'imah et al. (2023) | Indonesia | Caring burden among family caregivers of stroke patients in hospitals | To describe the burden experienced by family caregivers in caring for stroke patients | Semi-structured interviews | To determine the level of burden experienced by family caregivers and examine relationships with caregiver and patient characteristics | Structured survey | Zarit Burden Interview questionnaire (Bédard et al. 2001) |
| Parkinson et al. (2022) | UK | Care partnerships (e.g., spouses, caregivers) experiencing anxiety and depression symptoms post-stroke, using an online mindfulness intervention | To explore experiences and views of stroke survivors and their care partners using online Mindfulness-based Intervention (MBI) | Semi-structured, in-depth interviews | To investigate clinical effectiveness and outcomes of online mindfulness-based intervention (MBI) in reducing anxiety, depression, and improving mindfulness and mutuality among stroke survivors and care partners | Pre/post-test within-subject case study design | Hospital Anxiety Depression Scale (HADS; Zigmond & Snaith, 1983); Mutuality Scale (MS; Archbold et al. 1990); Mindful Attention Awareness Scale (MAAS; Brown & Ryan, 2003) |
| Rahman et al. (2024) | Malaysia | Coping with caregiving burden in a community-based stroke rehab context | To explore coping strategies among family caregivers of stroke survivors | Semi-structured interviews | To determine the coping strategies employed by stroke caregivers | Structured survey | Coping Strategies Inventory (Tobin et al. 1984) |
| Viscogliosi et al. (2019) | Canada | Family caregivers' role in supporting stroke survivors in relation to their caregiving burden | To examine the types of help caregivers provide to support older stroke survivors' participation | Semi-structured interviews at 1, 3, and 6 months | To determine how these types of help varied according to the caregiver's burden level (different burden dimensions) | Structured questionnaire at same timepoints | Caregivers' Perceived Burden Scale (Dumont et al. 1998). Cognitive status tests (e.g., WMS-III Logical Memory, MVPT-V, Stroop), and social participation with the Assessment of Life Habits (LIFE-H 3.1) |
| Ye et al. (2024) | China | Development and influencing factors of family resilience during stroke recovery | To understand the adaptation process of family resilience among stroke survivors and their caregivers | Semi-structured, in-depth interviews | To identify and examine direct and indirect relationships among family resilience, perceived social support, self-efficacy, and self-perceived burden | Surveys | Perceived Social Support Scale (Jiang, 2001); Stroke Self-Efficacy Questionnaire (Li et al. 2015), Self-Perceived Burden Scale (Wu & Jiang, 2010), Family Hardiness Index (Liu et al. 2014) |
| Yousaf et al. (2019) | Pakistan | Caregiver needs, challenges, and stressors | To explore the needs and caregiving experiences of high-risk caregivers of stroke patients | In-depth interviews | To identify high-risk caregivers and assess the association between their risk status and caregiving burden or related factors | Structured questionnaire | Structured pre-tested questionnaire (Caregiver Risk Screen Tool) used to classify caregivers by risk level |

*Note:* Full references for standardised instruments listed in the “Quantitative scales used” column are provided below.

Antonovsky, A. (1993). The structure and properties of the sense of coherence scale. *Social Science & Medicine, 36*(6), 725–733. <https://doi.org/10.1016/0277-9536(93)90033-Z>

Archbold, P. G., Stewart, B. J., Greenlick, M. R., & Harvath, T. (1990). Mutuality and preparedness as predictors of caregiver role strain. *Research in Nursing & Health*, *13*, 315–324.

Bakas, T., & Champion, V. (1999). Development and psychometric testing of the Bakas Caregiving Outcome Scale. *Nursing Research, 48,* 250–259.

Barrera, M., Sandler, I., & Ramsay, T. B. (1981). Preliminary development of a scale of social support: Studies on college students. *American Journal of Community Psychology, 9*(4), 435–447. <https://doi.org/10.1007/BF00918174>

Bédard, M., Molloy, D. W., Squire, L., Dubois, S., Lever, J. A., & O’Donnell, M. (2001). The Zarit Burden Interview: A new short version and screening version. The Gerontologist, 41(5), 652–657. https://doi.org/10.1093/geront/41.5.652

Benedict, R. H. B., Schretlen, D., Groninger, L., & Brandt, J. (1998). Hopkins Verbal Learning Test—Revised: Normative data and analysis of inter-form and test–retest reliability. *The Clinical Neuropsychologist, 12*(1), 43–55. <https://doi.org/10.1076/clin.12.1.43.1726>

Biener, L., & Abrams, D. B. (1991). The contemplation ladder: Validation of a measure of readiness to consider cessation. *Health Psychology, 10*(5), 360–365. <https://doi.org/10.1037/0278-6133.10.5.360>

Blesa, R. (2000). Galantamine: Therapeutic effects beyond cognition. *Dementia and Geriatric Cognitive Disorders, 11*(Suppl. 1), 28–34.

Cameron, J. I., Franche, R.-L., Cheung, A. M., & Stewart, D. E. (2002). Lifestyle interference and emotional distress in family caregivers of advanced cancer patients. *Cancer, 94*(2), 521–527. <https://doi.org/10.1002/cncr.10212>

Cameron, J. I., Cheung, A. M., Streiner, D. L., Coyte, P. C., & Stewart, D. E. (2006). Stroke survivors’ behavioral and psychological symptoms are associated with informal caregivers’ experiences of depression. *Archives of Physical Medicine and Rehabilitation, 87*(2), 177–183. <https://doi.org/10.1016/j.apmr.2005.10.012>

Clipp, E. C., & Moore, M. J. (1995). Caregiver time use: An outcome measure in clinical-trial research on Alzheimer’s disease. Clinical Pharmacology & Therapeutics, 58(2), 228–236.

Creswell, J. W., & Plano Clark, V. L. (2018). *Designing and conducting mixed methods research* (3rd ed.). Sage Publications.

de Man-van Ginkel, J. M., Hafsteinsdóttir, T., Lindeman, E., Burger, H., Grobbee, D., & Schuurmans, M. (2012). An efficient way to detect post-stroke depression by subsequent administration of a 9-item and a 2-item patient health questionnaire. *Stroke, 43*(3), 854–856. <https://doi.org/10.1161/STROKEAHA.111.640276>

Dumont, C., St-Onge, M., Fougeyrollas, P., & Renaud, L. (1998). Le fardeau perçu par les proches de personnes ayant des incapacités physiques [The burden perceived by relatives of people with physical disabilities]. *Canadian Journal of Occupational Therapy, 65*(5), 258–270.

D’Zurilla, T. J., & Sheedy, C. F. (1991). Relation between social problem-solving ability and subsequent level of psychological stress in college students. *Journal of Personality and Social Psychology, 61*(5), 841–846. <https://doi.org/10.1037/0022-3514.61.5.841>

D’Zurilla, T. J., Nezu, A. M., & Maydeu-Olivares, A. (1996). *Social Problem-Solving Inventory–Revised*. State University of New York at Stony Brook.

D’Zurilla, T. J., Nezu, A. M., & Maydeu-Olivares, A. (2002). *Social problem-solving inventory–revised (SPSI-R): Technical manual*. Multi-Health Systems.

Elmståhl, S., Malmberg, B., & Annerstedt, L. (1996). Caregiver’s burden of patients 3 years after stroke assessed by a novel caregiver burden scale. *Archives of Physical Medicine and Rehabilitation, 77*(2), 177–182.

Epstein, N. B., Baldwin, L. M., & Bishop, D. S. (1983). The McMaster Family Assessment Device. *Journal of Marital and Family Therapy, 9,* 77Y84.

Etter, J. F., Humair, J. P., Bergman, M. M., & Perneger, T. V. (2000). Development and validation of the attitudes towards smoking scale (ATS-18). *Addiction, 95*(4), 613–625.

Fetters, M. D., Curry, L. A., & Creswell, J. W. (2013). Achieving integration in mixed methods designs principles and practices. *Health Services Research*, 48(6), 2134–2156.

Fugl-Meyer, A. R., Melin, R., & Fugl-Meyer, K. S. (2002). Life satisfaction in 18- to 64-year-old Swedes: In relation to gender, age, partner and immigrant status. *Journal of Rehabilitation Medicine, 34*(5), 239–246. <https://doi.org/10.1080/165019702760279242>

Granger, C. V., Hamilton, B. B., & Sherwin, F. S. (1986). Guide for the use of the uniform data set for medical rehabilitation. Project Office, Department of Rehabilitation Medicine, Buffalo General Hospital.

Greene, J. C., Caracelli, V. J., & Graham, W. F. (1989). Toward a conceptual framework for mixed-method evaluation designs. *Educational Evaluation and Policy Analysis*, *11*(3), 255–274.

Jiang, Q. (2001). Perceived Social Support Scale. *Chinese Journal of Behavioral Medicine and Brain Science, 10*(1), 41–43.

Jordan, M. M., Freytes, I. M., Orozco, T., Tuan, A. W., Dang, S., Rutter, T., & Uphold, C. R. (2022). The RESCUE problem solving intervention for stroke caregivers: A mixed-methods pilot study. *Rehabilitation Psychology*, *67*(4), 484–496. https://doi.org/10.1037/rep0000460

Katz, S., Ford, A. B., Moskowitz, R. W., Jackson, B. A., & Jaffe, M. W. (1963). Studies of illness in the aged: The index of ADL: A standardized measure of biological and psychosocial function. *JAMA, 185*, 914–919. <https://doi.org/10.1001/jama.1963.03060120024016>

Krause, N. (1995). Negative interaction and satisfaction with social support among older adults. *The Journals of Gerontology: Series B, Psychological Sciences and Social Sciences, 50*(2), P59–P73.

Krause, N., & Markides, K. (1990). Measuring social support among older adults. *International Journal of Aging & Human Development, 30*(1), 37–53. <https://doi.org/10.2190/CY26-XCKW-WY1V-VGK3>

Lai, S., Perera, S., Duncan, P. W., & Bode, R. (2003). Physical and social functioning after stroke: Comparison of the Stroke Impact Scale and Short Form-36. *Stroke, 34*(2), 488–493. <https://doi.org/10.1161/01.STR.0000054162.94998.C0>

Liu, Y., Yang, J., Ye, B., Shen, Q., Zhu, J., & Chen, M. (2014). Reliability and validity of the Chinese version of the Family Hardiness Index. *Chinese Journal of Nursing Administration, 14*(7), 770–772.

Li, H., Fang, L., Bi, R., Cheng, H., Huang, J., & Feng, L. (2015). The reliability and validity of the Chinese version of Stroke Self-Efficacy Questionnaire. *Chinese Journal of Nursing, 50*(7), 790–794.

Mahoney, F. I., & Barthel, D. W. (1965). Functional evaluation: The Barthel Index. *Maryland State Medical Journal, 14*, 61–65.

McNair, D. M., Lorr, M., & Droppleman, L. F. (1992). *Manual for the Profile of Mood States–Revised.* Educational and Industrial Testing Service.

Radloff, L. S. (1977). The CES-D scale: A self-report depression scale for research in the general population. *Applied Psychological Measurement, 1*(3), 385–401. <https://doi.org/10.1177/014662167700100306>

Rohsenow, D. J., Martin, R. A., Tidey, J. W., Monti, P. M., & Colby, S. M. (2013). Comparison of the cigarette dependence scale with four other measures of nicotine involvement: Correlations with smoking history and smoking treatment outcome in smokers with substance use disorders. *Addictive Behaviors, 38*(9), 2409–2413.

Sherbourne, C. D., & Stewart, A. L. (1991). The MOS Social Support Survey. *Social Science & Medicine, 32*(6), 705–714. <https://doi.org/10.1016/0277-9536(91)90150-B>

Spek, V., Lemmens, F., Chatrou, M., van Kempen, S., Pouwer, F., & Pop, V. (2013). Development of a smoking abstinence self-efficacy questionnaire. *International Journal of Behavioral Medicine, 20*(3), 444–449.

Stalvey, B. T., Owsley, C., Sloane, M. E., & Ball, K. (1999). The Life Space Questionnaire: A measure of the extent of mobility of older adults. *Journal of Applied Gerontology, 18*(4), 460–478. <https://doi.org/10.1177/073346489901800404>

Steffen, A. M., McKibbin, C., Zeiss, A. M., Gallagher-Thompson, D., & Bandura, A. (2002). The revised scale for caregiving self-efficacy: Reliability and validity studies. *The Journals of Gerontology: Series B, Psychological Sciences and Social Sciences, 57*(1), P74–P86. <https://doi.org/10.1093/geronb/57.1.P74>

Sullivan, K., & Dunton, N. J. (2004). Development and validation of the Stroke Knowledge Test. *Topics in Stroke Rehabilitation, 11*(2), 19–28.

Tobin, D. L., Holroyd, K. A., & Reynolds, R. V. C. (1984). *User’s manual for the Coping Strategies Inventory*. Ohio University, Department of Psychology.

Toseland, R. W., Rossiter, C. M., & Labrecque, M. D. (1989). The effectiveness of peer-led and professionally led groups to support family caregivers. The Gerontologist, 29, 465–471.

Ware, J. E., Snyder, M. K., Wright, W. R., & Davies, A. R. (1983). Defining and measuring patient satisfaction with medical care. *Evaluation and Program Planning, 6*(3–4), 247–263. <https://doi.org/10.1016/0149-7189(83)90005-8>

Ware, J. M., & Keller, S. (1994). *SF-36 physical and mental health summary scales: A user’s manual*. The Health Institute.

Watson, D., Clark, L. A., & Tellegen, A. (1988). Development and validation of brief measures of positive and negative affect: The PANAS scales. *Journal of Personality and Social Psychology, 54*(6), 1063–1070. <https://doi.org/10.1037/0022-3514.54.6.1063>

Weathers, F. W., Litz, B. T., Huska, J. A., & Keane, T. M. (1994). *PTSD Checklist – Civilian Version. U.S*. Department of Veterans Affairs. http://www.mirecc.va.gov/docs/visn6/3_PTSD_CheckList_and_Scoring.pdf

Wu, Y., & Jiang, Y. (2010). Investigation and analysis of the self-perceived burden among cancer patients. *Journal of Nursing Administration, 10*(7), 405–407.

Yesavage, J. A., Brink, T. L., Rose, T. L., Lum, O., Huang, V., Adey, M., & Leirer, V. O. (1983). Development and validation of a geriatric depression screening scale: A preliminary report. *Journal of Psychiatric Research, 17*(1), 37–49. <https://doi.org/10.1016/0022-3956(82)90033-4>
